# Multi-omics reveal vitamin D regulation of immune-gut microbiome interactions and tolerogenic pathways in inflammatory bowel disease

**DOI:** 10.1016/j.xcrm.2026.102703

**Published:** 2026-03-26

**Authors:** John Gubatan, Raoul S. Sojwal, Jiayu Ye, Theresa L. Boye, Jacqueline N. Hoang, Touran Fardeen, Michelle Temby, Samuel J.S. Rubin, Sean P. Spencer, Prasanti Kotagiri, Stephan Rogalla, Michael J. Rosen, Ole Haagen Nielsen, Scott Boyd, Justin Sonnenburg, Sidhartha R. Sinha

**Affiliations:** 1 Division of Gastroenterology and Hepatology, Mayo Clinic, Jacksonville, FL 32224, USA; 2 Division of Gastroenterology and Hepatology, Stanford University School of Medicine, Stanford, CA 94305, USA; 3 Department of Pathology, Stanford University, Stanford, CA 94304, USA; 4 Microbiology & Immunology, Stanford School of Medicine, Stanford, CA 94305, USA; 5 Division of Pediatric Gastroenterology, Hepatology, and Nutrition, Stanford University School of Medicine, Stanford, CA 94304, USA; 6 Department of Gastroenterology, Medical Section, Herlev Hospital, University of Copenhagen, 2730 Herlev, Denmark; 7 Department of Immunology and Pathology, Monash University, Melbourne 3004, VIC, Australia; 8 Center for Human Microbiome Studies, Stanford University, Stanford, CA 94305, USA; 9 Sean N. Parker Center for Allergy & Asthma Research, Stanford University, Stanford, CA 94304, USA

**Keywords:** inflammatory bowel disease, gut microbiome, immune tolerance, vitamin D, immune repertoire

## Abstract

Loss of immune tolerance to the gut microbiome plays a pathogenic role in inflammatory bowel disease (IBD). How dietary factors alter host immune-gut microbiome interactions in IBD is unclear. Here, we apply multi-omics (immunoglobulin A or G and 16S rRNA sequencing [IgA-seq, IgG-seq], blood single-cell RNA sequencing [scRNA-seq], and immune repertoire sequencing) to investigate the effects of 12 weeks of vitamin D on host immune microbe interactions in patients with IBD. Vitamin D treatment associates with decreased disease activity and inflammatory markers and increased IgA-bound and decreased IgG-bound gut microbiota. Vitamin D alters the profiles of IgA-bound (increased *Lachnospiraceae*, *Blautia*) and IgG-bound (decreased *Proteobacteria*, *Enterococcaceae)* gut bacteria. Vitamin D increases B cell activating factor (BAFF) signaling between plasmacytoid dendritic cells and B cells, alters BCR and TCR clonotypes that associate with Ig-bound gut microbiota, and increases α4β7+ B and T regulatory cells. Our results demonstrate that vitamin D promotes immune tolerance to gut microbiota in patients with IBD. Clinical trial is registered under NCT04828031.

## Highlights

• Vitamin D increases IgA- and decreases IgG-bound gut microbiota in inflammatory bowel disease
• Vitamin D alters Ig-bound microbiota profiles and immune repertoire-gut microbiota networks
• Vitamin D induces BAFF signaling between plasmacytoid dendritic cells and B cells
• Vitamin D increases gut tropic α4β7+ CX3CR1 B regulatory and T regulatory cells

## Graphical abstract

**Figure:**
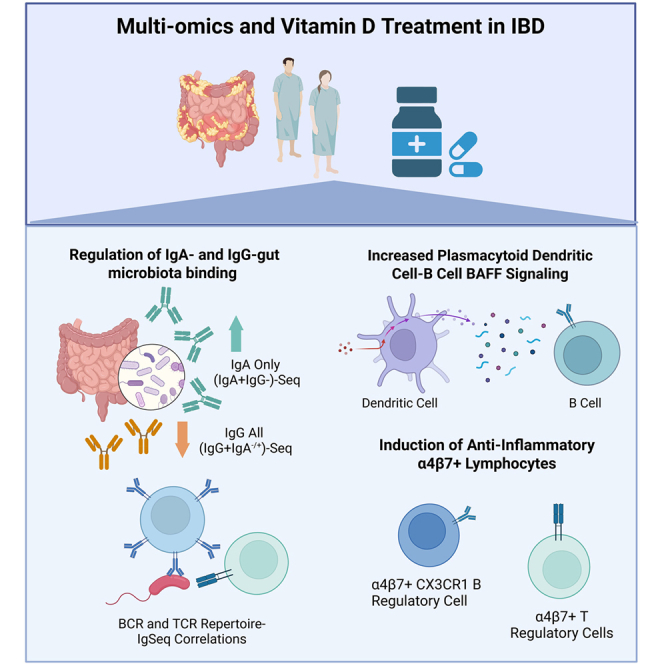

## Teaser

Gubatan et al. demonstrate vitamin D’s immunoregulatory role on host immune-microbe interactions in patients with inflammatory bowel disease. Vitamin D alters the profiles of IgA- and IgG-bound gut bacteria and immune repertoire-gut microbiota networks, induces plasmacytoid dendritic cell-B cell BAFF signaling, and increases α4β7+ B and T regulatory cells.

## Introduction

Inflammatory bowel disease (IBD), which includes ulcerative colitis and Crohn disease, is a chronic inflammatory disorder of the gastrointestinal tract that is thought to arise from a complex interplay between host genetic predisposition^1^ and environmental triggers.^2^ Loss of immune tolerance to commensal gut bacteria has been recognized to play a pivotal role in the pathogenesis of IBD.^3^ Current therapeutic strategies in IBD have focused mainly on targeting dysregulated immune responses^4^ without directly addressing gut microbiome crosstalk with intestinal immunity. Understanding the mechanisms that regulate host immune-microbe interactions and developing ways to restore immune tolerance to gut microbiota could lead to therapeutic strategies to treat or prevent IBD.

Immunoglobulin A (IgA) is secreted as a dimeric antibody at mucosal surfaces including the gastrointestinal tract.^5^^,^^6^ IgA binds to a wide range of commensal gut bacteria,^7^^,^^8^ plays a critical role in protecting against enteric infections, and regulates gut microbiota composition and symbiosis to maintain intestinal immune homeostasis.^9^^,^^10^ IgA class switching occurs through T-cell-independent (via secretion of B cell activating factor [BAFF] and a proliferation-inducing ligand [APRIL] by dendritic cells) and T-cell-dependent (through transforming growth factor β [TGF-β] and CD40 ligand [CD40L] expression by CD4 T cells) mechanisms.^11^ IgA and IgG binding to gut bacteria is increased in patients with IBD and correlates with disease activity and inflammation.^12^^,^^13^ Prior work has revealed that profiles of IgA-bound gut bacteria are distinct in new-onset IBD, are associated with time to surgery in patients with IBD,^14^ and target colitogenic bacteria.^15^ IgG-bound gut bacteria are increased in ulcerative colitis and propagate inflammation by engagement of Fcγ receptors (FcγRs) on gut-resident macrophages and subsequent activation of downstream interleukin (IL)-1β-dependent type 17 signaling.^16^ Furthermore, IgG-bound gut bacteria may represent translocating gut bacteria or targets of systemic immunity in patients with IBD.^17^ Clinical strategies to manipulate IgA and IgG binding to gut microbiota in IBD to restore immune tolerance are lacking and limited in part by an incomplete understanding of their regulation.

Higher vitamin D levels and vitamin D supplementation have been associated with gut microbiome composition shifts toward increased diversity and enrichment of beneficial gut bacteria in healthy adults^18^^,^^19^^,^^20^ and in patients with IBD.^21^^,^^22^^,^^23^ Furthermore, vitamin D has potent immunomodulatory effects on both B and T cells.^24^ Vitamin D can decrease B cell proliferation, plasma cell differentiation, and IgM and IgG secretion,^25^^,^^26^ which may be mediated indirectly through the effects on dendritic cells and T helper cells.^27^ Vitamin D can attenuate inflammatory T cell responses (T_H_1 and T_H_17) and induce differentiation of FOXP3+ T regulatory cells.^28^^,^^29^ Vitamin D has also been associated with expression of the gut tropic integrin α4β7 (plays role in trafficking to the gastrointestinal tract) on immune cells^30^^,^^31^ including B cells.^32^

Given the role of vitamin D at the interface between gut microbiome, B cell immunity and antibody production, and gut trafficking integrin α4β7, we hypothesized that vitamin D regulates immunoglobulin binding to commensal gut bacteria and α4β7+ B cell immunophenotypes in patients with IBD. To test this hypothesis, we performed an interventional study of vitamin D treatment (NCT04828031) in patients with IBD and low vitamin D. Here, we studied the effects of vitamin D on IgA and IgG binding to gut bacteria using bacterial fluorescence-activated cell sorting, 16S sequencing (IgA-seq, IgG-seq), and effects on peripheral blood immune response using single-cell transcriptomics (scRNA-seq) and B cell receptor (BCR) and T cell receptor (TCR) repertoire sequencing. Our study revealed that vitamin D was associated with decreased disease activity and inflammatory markers and increased IgA binding and decreased IgG binding to gut microbiota. Vitamin D had differential effects on Ig-bound gut bacterial taxa including increased IgA-bound f_*Lachnospiraceae* and *g_Blautia* and decreased IgG-bound p_*Proteobacteria* and *g_Enterococcaceae*. Vitamin D also led to increased BAFF signaling between plasmacytoid dendritic cells (pDCs) and B cells, differentially altered BCR and TCR clonotypes associated with specific Ig-bound gut microbiota, and increased α4β7+ CX3CR1 B regulatory and T regulatory cells. Taken together, our results provide insights into host immune-microbe interactions and the vitamin D regulation of immune tolerance to gut microbiota in patients with IBD.

## Results

### Vitamin D is associated with improved disease activity and stool inflammatory marker in inflammatory bowel disease clinical trial

**Figure 1:**
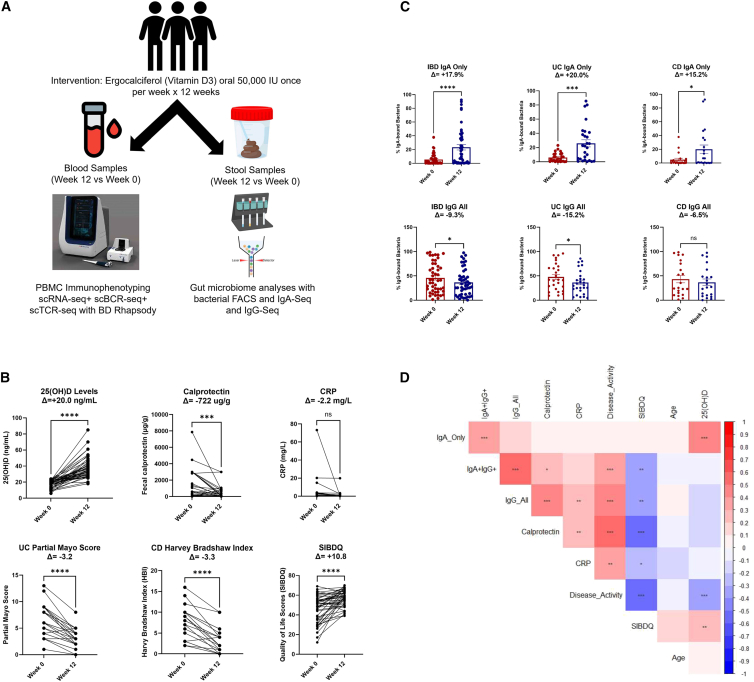
Study schematic and clinical trial outcomes in IBD (A) Vitamin D clinical trial, sample collection, and processing overview. (B) Vitamin D treatment leads to improvement in serum vitamin D (25(OH)D) levels, fecal calprotectin, disease activity scores (partial Mayo scores and Harvey Bradshaw index), and quality-of-life scores (SIBDQ). Data are represented as mean ± SEM. (C) Vitamin D increased IgA-only binding while decreasing IgG-all binding to the gut microbiota in patients with IBD. Data are represented as mean ± SEM. (D) Correlation matrix demonstrating association of IgA-bound and IgG-bound gut microbiota with IBD clinical parameters. Stars indicate nominal Wilcoxon signed-rank test *p* values: ns: *p* > 0.05; ∗*p* < 0.05; ∗∗*p* < 0.01; ∗∗∗*p* < 0.01.

Forty-eight patients with IBD completed the clinical trial and provided a full set of pre- and post-vitamin D treatment blood and stool samples (study schematic summarized in Figure 1A) and are included in the final analysis. Table 1 summarizes the baseline clinical characteristics of patients in the clinical trial. The mean age of patients was 38.96 years, and 45.8% were male and 54.2% were female. About 56.3% of patients had ulcerative colitis (UC), whereas 43.7% had Crohn disease (CD). The mean serum vitamin D [25(OH)D] level was 18 ng/mL, and mean fecal calprotectin was 1,046.3 μg/g. In terms of pre-treatment IBD therapies, 41.7% patients were on anti-TNF, 10.4% on anti-α4β7, and 8.3% on anti-IL12/23 biologic therapies. Six patients had medication changes (two steroids and four anti-TNF agents) within 4 weeks prior to starting the vitamin D trial. No patients had any medication changes during the 12-week vitamin D treatment period. Twelve weeks of 50,000 units of oral vitamin D once per week led to a 20 point increase in serum 25(OH)D levels (*p* < 0.0001), a decrease in fecal calprotectin by 722 μg/g (*p* < 0.001), a decline in disease activity scores (partial mayo score for ulcerative colitis, −3.2, *p* < 0.0001; Harvey Bradshaw Index for Crohn disease, −3.3, *p* < 0.0001), and a 10.8 increase in quality of life scores (measured by SIBDQ, *p* < 0.0001). Vitamin D did not significantly affect blood C-reactive protein (CRP) levels (Figure 1B).

**Table 1:** Baseline clinical characteristics of patients with inflammatory bowel disease

**Demographics**	

Age (mean years ± SD)	38.96 ± 13.13
Male	22 (45.8)
Female	26 (54.2)

**Diagnosis**	

Ulcerative colitis (UC), N (%)	27 (56.3)
Disease Location	
E1 – proctitis	1 (2.1)
E2 – left-sided	3 (6.3)
E3 – pancolitis	23 (47.9)
Crohn disease (CD), N (%)	21 (43.7)
Disease Location	
L1 – ileal	3 (6.3)
L2 – colonic	1 (2.1)
L3 – ileocolonic	17 (35.4)
L4 – upper GI tract	0 (0)

**Labs and Disease Activity**	

Mean 25(OH)D (ng/mL)	18
Mean fecal calprotectin (μg/g)	1046.3
Mean C-reactive protein (mg/L)	3
Mean Partial Mayo Score	6.5
Mean Harvey Bradshaw Index	6.5

**Medications, N (%)**	

None	2 (4.2)
Mesalamine	13 (27.1)
Prednisone	1 (2.1)
Anti-α4β7	5 (10.4)
Anti-TNF	20 (41.7)
Anti-IL-12/23	4 (8.3)
Immunomodulators^a^	0 (0)
Antibiotics	0 (0)
Med changes before vitamin D^b^	6 (12.5)
Med changes during vitamin D	0 (0)

a Immune modulators include 6-mercaptopurine, azathioprine, and methotrexate.

b Medication changes (two on prednisone, three on infliximab, and one on adalimumab).

### Vitamin D differentially regulates IgA- and IgG-bound gut microbiota composition and inferred metagenome function

Vitamin D was associated with an increase in levels of secretory IgA from stool supernatants (+744.6 ng/mL, *p* < 0.01) and serum IgA (+0.90 ng/mL, *p* < 0.01). There was a non-significant decrease in fecal IgG levels (−279.5 ng/mL, *p* = 0.18) and no difference in serum IgG levels (*p* = 0.77) after vitamin D treatment (Figures S1A and S1B). Vitamin D led to increased IgA-only (IgA+IgG−) binding to gut bacteria (+17.9%, *p* < 0.001) and decreased IgG-all (combined IgG+IgA− and IgG+IgA+) binding to gut bacteria (−9.3%, *p* < 0.05). In subgroup analyses according to IBD subtype, vitamin D led to a 20% increase in IgA-only bound gut bacteria in ulcerative colitis (*p* < 0.01) and 15.2% increase in Crohn disease (*p* < 0.05). Vitamin D led to a 15.2% decrease in IgG-all binding to gut bacteria in ulcerative colitis (*p* < 0.05) and a nonsignificant decrease by 6.5% in Crohn disease (Figure 1C). In sensitivity analyses to test for the confounding effects of medications, there were no differences in IgA-bound and IgG-bound gut bacteria among patients on different IBD medication classes (Table S1; Figures S2A and 2B). In correlation analyses, IgA-only bound gut bacteria correlated with serum 25(OH)D levels (Pearson rho = 0.408, *p* < 0.0001), while IgG-all bound gut bacteria correlated with disease activity (Pearson rho = 0.447, *p* < 0.0001) and fecal calprotectin (Pearson rho = 0.439, *p* < 0.0001) (Figure 1D). Vitamin D did not result in any significant changes in alpha diversity via Shannon (Kruskal-Wallis, *p* = 0.48) (Figure S3A) or beta diversity (Bray Curtis dissimilarity, R2 = 0.03, PERMANOVA, *p* = 0.32) after adjusting for inflammation status (Figure S3B). In terms of whole gut microbiome composition, vitamin D led to increased abundance (Lefse LDA >3, Wilcoxon *p* < 0.05) of *f_Ruminococcaceae*, *g_Faecalibacterium*, *s_prausnitzii*, *s_biforme*, *s_Roseburia*, *g_Oscillospira*, and *g_Lachnospira* and decreased abundance in *f_Enterobacteriaceae*, *f_Lactobacillaceae*, *g_Pediococcis*, *s_zeae*, *g_Corynebacterium*, and *f_Burkholderiaceae* (Figure S3C).

**Figure 2:**
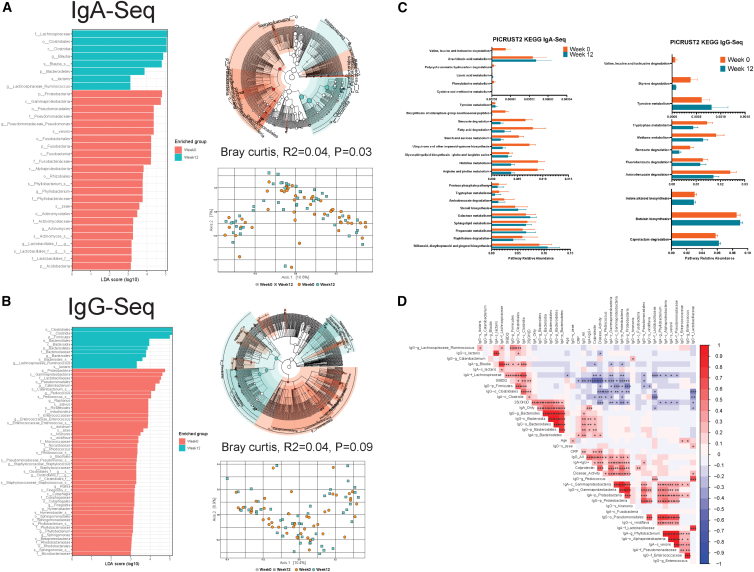
Vitamin D differentially regulates composition and predicted metagenome function of IgA- and IgG-bound gut microbiota (A) IgA-seq reveals IgA-bound gut microbiota taxa significantly enriched after 12 weeks of vitamin D (*n* = 48 patients samples, two time points) by LEfSe linear discriminant analysis (LDA) effect size bar plot (left), LEfSe cladogram (top right), and Bray curtis ordination plot (bottom right). (B) IgG-seq reveals IgA-bound gut microbiota taxa significantly enriched after 12 weeks of vitamin D by LEfSe linear discriminant analysis (LDA) effect size bar plot (left), LEfSe cladogram (top right), and Bray curtis ordination plot (bottom right). (C) PICRUSt2 (Phylogenetic Investigation of Communities by Reconstruction of Unobserved States) predicts significant functional abundance differences of metagenomes in IgA-bound (left) and IgG-bound (right) gut microbiota after vitamin D. Data are represented as mean ± SEM. (D) Correlation matrix demonstrating association of top differentially abundant IgA-bound and IgG-bound microbiota taxa with IBD clinical parameters. Stars indicate nominal Wilcoxon signed-rank test *p* values: ns: *p* > 0.05; ∗*p* < 0.05; ∗∗*p* < 0.01; ∗∗∗*p* < 0.01.

Vitamin D did not lead to any significant changes in alpha diversity via Shannon index in both IgA-bound (Kruskal-Wallis, *p* = 0.48) (Figure S3D) and IgG-bound (Kruskal-Wallis, *p* = 0.48) gut bacteria. After multivariate adjustment for inflammation, vitamin D led to a significant change in composition of IgA-bound gut bacteria (Bray Curtis dissimilarity, R2 = 0.04, PERMANOVA, *p* = 0.03) (Figure 2A). In differential abundance analysis with Lefse (LDA>3, Wilcoxon *p* < 0.05), vitamin D led to increased IgA binding to *f_Lachnospiraceae*, *o_Clostridiales*, *g_Blautia*, *p_Bacteroidetes*, *s_lactaris*, and *g_Lachnospiraceae_Ruminococcus*. Conversely, vitamin D led to decreased IgA binding to several taxa including *p_Proteobacteria*, *o_Pseudomonadales*, *s_veronii*, *p_Fusobacteria*, *s_zeae*, *f_Actinomycetaceae*, *f_Lactobacillales*, and *p_Acidobacteria* (Figure 2A).Likewise, using Palm Index, vitamin D was associated with increased IgA binding to *p_Firmicutes*, *f_Lachnospiraceae*, *o_Clostridiales*, and *g_Blautia* and decreased binding to *p_Proteobacteria* (Figure S4). Similarly, using IgA-seq probability ratio, vitamin D was associated with increased IgA binding to *p_Firmicutes*, *f_Lachnospiraceae*, *o_Clostridiales*, and *g_Blautia* and decreased binding to *p_Proteobacteria*, *p_Bacteroidetes*, and *p_Fusobacteria* (Figure S5). For IgG-bound gut bacteria, there was a trend toward a difference in gut bacteria community composition (Bray Curtis dissimilarity, R2 = 0.04, PERMANOVA, *p* = 0.09) (Figure 2B). In differential abundance analysis with Lefse (LDA>3, Wilcoxon, *p* < 0.05), vitamin D led to increased IgG binding to *o_Clostridiales*, *p_Firmicutes*, *f_Bacteroidaceae*, *g_Lachnospiraceae_Ruminococcus*, and *s_lactaris*. Conversely, vitamin D led to decreased IgG binding to several taxa including *p_Proteobacteria*, *o_Pseudomonadales*, *f_Lactobacillaceae*, *g_Pediococcus*, *f_Enterococcaceae*, *s_dolichum*, *s_hiranonis*, and *s_zeae* (Figure 2B). Likewise, using Palm Index, vitamin D was associated with decreased IgG binding to *p_Proteobacteria* and *f_Lactobacillaceae* (Figure S6). Similarly, using IgG-seq probability ratio, vitamin D was associated with decreased IgG binding to *p_Proteobacteria*, *p_Bacteroidetes*, *g_Enterococcus*, *f_Lactobacillaceae*, *and g_Pediococcus* (Figure S7).

Given the significant alterations in taxa of IgA- and IgG-bound gut bacteria with vitamin D, we next sought to understand if vitamin D led to changes in inferred bacterial metagenome function (KEGG pathways) using PICRUST2 (Douglas et al., 2022). Vitamin D altered IgA-bound gut microbial amino acid metabolism (decreased valine, isoleucine, and isoleucine degradation; decreased metabolism of phenylalanine, cysteine, methionine histidine, arginine, proline, and tryptophan; and increased metabolism of tyrosine), carbohydrate metabolism (decreased metabolism of starch and sucrose, decreased pentose phosphate pathway, and increased galactose metabolism), fatty acid metabolism (decreased fatty acid degradation, increased short chain fatty acid propanoate metabolism), and increased stilbenoid, diarylheptanoid, and gingerol biosynthesis (Figure 2C). Likewise, vitamin D altered IgG-bound gut microbial amino acid metabolism (decreased valine, isoleucine, and isoleucine degradation; decreased metabolism of tryptophan; and increased metabolism of tyrosine), decreased methane metabolism, decreased benzoate degradation, and increased betalain biosynthesis and caprolactam degradation (Figure 2C).

To understand the clinical significance of several IgA- and IgG-bound gut bacteria, we performed correlation analyses (Figure 2D) to determine their associations with clinical parameters in patients with IBD. Several Ig-bound bacteria were positively associated with disease activity (*IgA-c_Gammaproteobacteria*, *IgA-p_Proteobacteria*, *IgG-p_Proteobacteria*, *IgG-g_Pediococcus*, *IgG-f_Lactobacillaceae*) and calprotectin, a stool biomarker for inflammation (*IgA-c_Gammaproteobacteria*, *IgA-p_Proteobacteria*, *IgA-c_Fusobacteria*, *IgG-c_Gammaproteobacteria*, *IgG-p_Proteobacteria*, *IgG-p_Bacteroidetes*, *IgA-p_Bacteriodetes*, *IgG-o_Bacteroidales*). Conversely, several Ig-bound gut bacteria were negatively associated with disease activity (*IgG-p_Firmicutes*, *IgG-s_lactaris*, *IgA-f_Lachnospiraceae*, *IgA-c_Clostridia*) and calprotectin *(IgA-c_Clostridia*, *IgA-g_Blautia*, *IgA-f_Lachnospiraceae*) (Figure 2D).

### Vitamin D alters PBMC composition and promotes BAFF signaling between plasmacytoid dendritic cells and B cells

**Figure 3:**
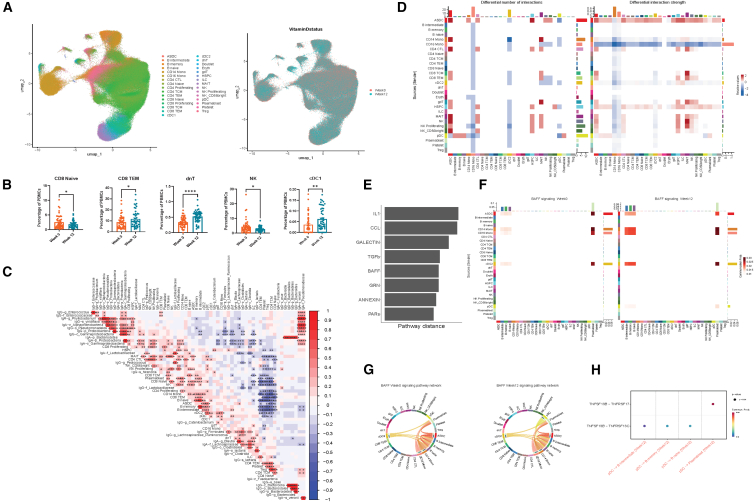
scRNA-seq reveals effects of vitamin D on peripheral blood mononuclear cell (PBMC) composition and signaling between dendritic cells and B cells in IBD (A) Uniform manifold approximation and projection (UMAP) of PBMC scRNA-seq (375K cells) dataset from vitamin D clinical trial patients (*n* = 48 patients samples, two time points) with all cell types (left), by vitamin D status (right). (B) Boxplot demonstrating PBMC cell types with significant change with vitamin D. Data are represented as mean ± SEM. (C–H) (C) Correlation matrix demonstrating immunogenicity of Ig-bound bacteria with PBMC subtypes. (D) *CellChat* heatmap demonstrates increased number and strength of cell-cell interactions between dendritic cells and B cell subtypes in week 12 vs. week 0 of vitamin D treatment. (E) Systems pathway analyses reveal B cell activating factor (BAFF) signaling as a top pathway most altered in week 12 vs. week 0 vitamin D. (F) Heatmap demonstrating increased BAFF signaling between dendritic cells and B cells after 12 weeks of vitamin D. (G) Chord diagram showing activation of BAFF signaling between plasmacytoid dendritic cells (pDCs) and B cells (H)TNFSF13B:TNFRSF13C and TNFSF13B: TNFRSF17 ligand-receptor interactions between pDCs and B cells. Stars indicate nominal Wilcoxon signed-rank test *p* values: ns: *p* > 0.05; ∗*p* < 0.05; ∗∗*p* < 0.01; ∗∗∗*p* < 0.01.

To understand how vitamin D alters host immune peripheral blood cells in patients with IBD, we performed scRNA-seq on peripheral blood mononuclear cells (PBMCs) obtained before and after vitamin D treatment. Our final PBMC scRNA-seq dataset after quality control resulted in 375K cells (Figure 3A), with all major immune cell lineages (B cells, T cells, monocytes, dendritic cells, platelets, and erythrocytes) represented. In differential abundance analyses (Figure 3B), 12 weeks of vitamin D led to an increase in double-negative T lymphocytes (dnT) (*p* < 0.0001), conventional type 1 dendritic cells (cDC1) (*p* < 0.01), and CD8 T effector memory cells (CD8 TEM) (*p* < 0.05), while decreasing CD8 naive T cells (*p* < 0.05) and natural killer (NK) cells (*p* < 0.05). To understand the immunogenicity of Ig-bound gut bacteria, we performed correlation analyses to determine their association with peripheral blood immune cells (Figure 3C). *IgA-p_Proteobacteria* were positively associated with NK proliferating, mucosal-associated invariant T (MAIT) cells, CD14 monocytes, CD4 proliferating, and CD4 CTL. *IgA-f_Lachnospiraceae* were positively associated with dnT, innate lymphoid cells (ILCs), and AXL + SIGLEC6 + dendritic cells (ASDCs). *IgG-g_Enterococcus* were positively associated with central memory CD8 T cells (CD8 TCM). *IgA-s_veronii* and *IgA-f_Pseudomonadaceae* were both positively associated with T regulatory cells. *IgG-c_Gammaproteobacteria* were negatively associated with gamma delta T cells (gdT).

We next used CellChat to understand the impact of vitamin D on cell-cell interactions and signaling pathways in patients with IBD. The number and strength of cell-cell interactions increased between pDCs and B cell subsets (B naive, B intermediate, B memory, and plasmablasts) in week 12 versus week 0 vitamin D (Figure 3D). The strength of cell-cell interactions between ASDC and B cell subsets was also increased in week 12 versus week 0 vitamin D. Systems pathway analyses revealed eight signaling pathways (IL-1, CCL, GALECTIN, TGFb, BAFF, GRN, ANNEXIN, and PARs) that were significantly altered in week 12 versus week 0 vitamin D (Figure 3E). Among these pathways, only the B cell activating factor signaling pathway localized to dendritic cells and B cells (Figure 3F). Further analyses revealed that BAFF signaling was increased between pDCs and B cell subsets in week 12 versus week 0 vitamin D (Figure 3G) and were mediated through TNFSF13B-TNFRSF13C ligand-receptor interactions between pDCs and B naive, B intermediate, and B memory cells and TNFSF13B-TNFRSF17 ligand-receptor interactions between pDCs and plasmablasts (Figure 3H).

### Vitamin D leads to shifts in peripheral blood BCR and TCR clonotypes that are associated with specific Ig-bound gut bacteria

**Figure 4:**
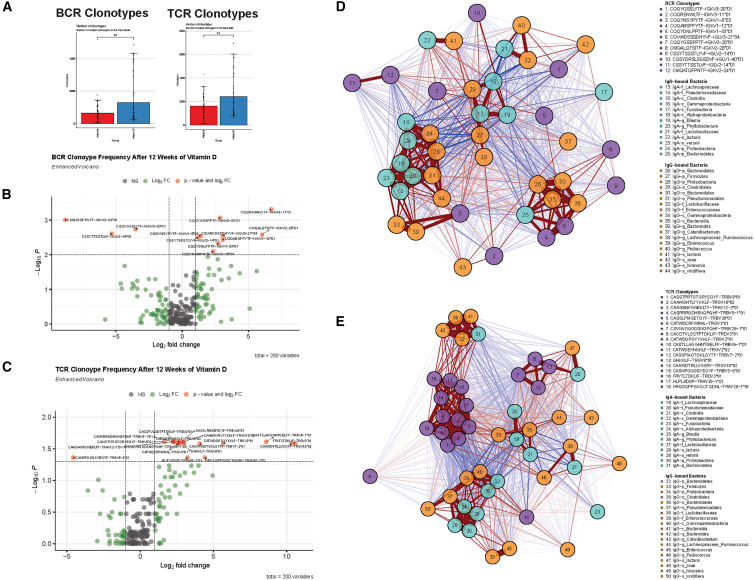
scBCR-seq and scTCR-seq reveal effects of vitamin D on immune repertoire and shared BCR and TCR clonotypes associated with Ig-bound gut microbiota (A–D) (A) Bar plot of number BCR clonotypes (left) and TCR clonotypes (right) of IBD patients according to vitamin D status (*n* = 48 patients, two time points). Data are represented as mean ± SEM. (B) Volcano plot showing significant BCR clonotypes altered with vitamin D among top 200 public BCR clonotypes in cohort. (C) Volcano plot showing significant TCR clonotypes altered with vitamin D among top 200 public TCR clonotypes in cohort. (D) Correlation networks demonstrating association of BCR clonotypes (purple nodes) with IgA- (turquoise nodes) and IgG-bound (orange nodes) gut microbiota taxa. (E) Correlation network plot demonstrating association of TCR clonotypes (purple nodes) with IgA- (turquoise nodes) and IgG-bound (orange nodes) gut microbiota taxa. Positive associations are denoted by red lines, and negative associations are denoted by blue lines. Weight of lines denote strength of correlations.

Given that vitamin D led to alterations in Ig binding to specific gut bacteria and promoted cellular interactions involved with immune tolerance and antigen presentation (dendritic cell-B cell interactions), we hypothesized that vitamin D could regulate BCR and TCR clonotypes that have reactivities to gut bacteria in IBD. In addition to scRNA-seq, we also performed paired scBCR-seq and scTCR-seq from pre- and post-vitamin D blood samples. The relative abundance of BCR and TCR clonotypes according to patient sample and clonotype group are summarized in Figures S8A and S9A, respectively. There was a trend toward an increased number of BCR clonotypes (*p* = 0.1) and TCR clonotypes (*p* = 0.2) with vitamin D (Figure 4A). There was a similar trend toward increased BCR and TCR clonal diversity (by Chao1) with vitamin D (Figures S8B and S9B). Vitamin D did not alter the relative abundance of large and hyperexpanded BCR (Figure S8C) and TCR (Figure S9C) clonotypes. Vitamin D increased IGHA1 isotype usage frequency (*p* = 0.02) but had no significant effects on usage of other isotypes (IGHA2, IGHG1, IGHG2, IGHG3, and IGHG4), BCR complementarity-determining region (CDR3) length, or BCR somatic hypermutation (SHM) rates (Figure S9D).

To determine the effects of vitamin D on BCR and TCR clonotypes, we performed differential abundance analyses of the top 200 public (shared) BCR (Table S2) and TCR clonotypes (Table S3) in the IBD patient clinical trial cohort. Vitamin D led to a differential change in 12 BCR clonotypes (CDR3 variable coding regions) (*p* < 0.01, false discovery rate [FDR] <0.1) (Figure 4B; Table S2), including increased CQQRSNWLYTF-IGKV3-11∗01 (FC = 6.73), CMQALQTSITF-IGKV2-28∗01 (FC = 6.03), CQSYDRSLSGSDVF-IGLV1-40∗01 (FC 5.94), CQVWDSSSDHYVF-IGLV3-21∗04 (FC 3.07), CQQANSFPYTF-IGKV1-12∗01 (FC 3.07), CQQYGSSPPTF-IGKV3-20∗01 (FC 2.86), CQQYDNLPPTF-IGKV1-33∗01(FC 2.73), CQQYNSYPYTF-IGKV1-5∗03 (FC 1.39), and CSSYTSSSTLYVF-IGLV2-14∗01(FC 1.19) and decreased CMQATQFPNTF-IGKV2-24∗01(FC -8.83), CSSYTTSSTLVF-IGLV2-14∗01(FC -5.31), and CQQYGSSLYTF-IGKV3-20∗01 (FC -3.52). Likewise, vitamin D led to a differential change in 18 TCR clonotypes (*p* < 0.01, FDR <0.05) (Figure 4C; Table S3), including increased FRVTLTDKLIF-TRDV3∗01(FC 10.59), CAAWDTRLLVGSRY-TRGV10∗02(FC 10.34), CASTLLAGGHNTGELFF-TRBV6-7∗01(FC 8.60), CATWDGPGYYKKLF-TRGV2∗01(FC 5.76), HRQSQPPGKQCFQDNL-TRAV26-1∗01(FC 4.41), CASSPIAGTGKLGYTF TRBV7-2∗01(FC 4.07), HLPLSDSP-TRAV26-1∗01(FC 3.23), GHKKLF-TRGV9∗01(FC 2.98), CAAWGHTLYYKKLF-TRGV10∗02(FC 2.96), CASSLPMQETQYF-TRBV28∗01(FC 2.83), CATWDCRFWRAL-TRGV3∗01(FC 2.64), CSVNVGQGDSNQPQHF-TRBV29-1∗01(FC 2.63), CATWDEHNKKLF-TRGV2∗02(FC 2.62), CACDTVLGDTPTDKLIF-TRDV2∗01(FC 2.36), CASRRRGDHSNQPQHF-TRBV5-1∗01(FC 2.26), CAMSANFGNEKLTF-TRAV12-3∗01(FC 2.26), and CASSTPRTGTGRYEQYF-TRBV9∗01(FC 1.66). Only one TCR clonotype, CASNPGQGSYEQYF-TRBV5-6∗01, was significantly decreased (FC = −4.55) with vitamin D.

We next performed correlation network analyses to determine the association of differentially expressed BCR and TCR clonotypes with IgA- and IgG-bound gut bacteria in patients with IBD (Figures S8D and S9D; Tables S4 and S5; Figures 4D and 4E). In correlation network analyses, CQQYNSYPYTF-IGKV1-5∗03 was positively associated with IgG-g_*Enterococcus*, IgG-f_*Enterococcaceae*, and IgA-f_*Lachnospiraceae*. Two BCR clonotypes (CMQALQTSITF-IGKV2-28∗01 and CQQANSFPYTF-IGKV1-12∗01) were strongly and positively associated with IgG- and IgA-bound taxa belonging to p_*Bacteriodetes*. CQSYDRSLSGSDVF-IGLV1-40∗01 was positively associated with IgA-g_*Blautia* and IgA-f_*Lachnospiraceae*. CQQYGSSLYTF-IGKV3-20∗01 was positively associated with IgG-s_*zeae*, IgG-g_*Lachnospiraceae_Ruminococcus*, and IgG-g_*Pediococcus* and negatively associated with IgA-f_*Lachnospiraceae* and IgG-p_*Firmicutes* (Figure 4D). Several TCR clonotypes were also significantly associated with Ig-bound gut bacteria. GHKKLF-TRGV9∗01 was positively associated with IgA-s_*lactaris*, IgG-s_*lactaris*, and IgG-o_*Clostridiales*. Four TCR clonotypes (CATWDEHNKKLF-TRGV2∗02, FRVTLTDKLIF-TRDV3∗01, CASTLLAGGHNTGELFF-TRBV6-7∗01, and CAAWDTRLLVGSRY-TRGV10∗02) were strongly and positively associated with IgG- and IgA-bound taxa belonging to p_*Bacteriodetes*. CASNPGQGSYEQYF-TRBV5-6∗01 was positively associated with IgA-c_*Gammaproteobacteria*, IgA-p_*Proteobacteria*, IgG-o_*Pseudomonadales*, IgG-c_*Gammaproteobacteria*, IgG-p_*Proteobacteria*, and IgA-f_*Lactobacillaceae* and negatively associated with IgA-f_*Lachnospiraceae* and IgA-c_*Clostridia*. CASSTPRTGTGRYEQYF-TRBV9∗01 was positively associated with IgG-g_*Pediococcus* and negatively associated with IgA- and IgG-bound taxa belonging to p_*Proteobacteria* (Figure 4E).

### scRNA-seq reveals α4β7+ B cell heterogeneity and induction of α4β7+ CX3CR1 B regulatory cells with vitamin D

We next focused our analyses on immune cells that traffic to the gastrointestinal tract through expression of the gut tropic integrin α4β7. We created a subset of α4β7+ cells from our main PBMC scRNA-seq dataset, which yielded 163.6K cells (Figure S10A). In differential abundance analyses, α4β7+ cDC1, dnT, and ILCs were increased, while α4β7+ NK cells were decreased with vitamin D (Figure S10B).

**Figure 5:**
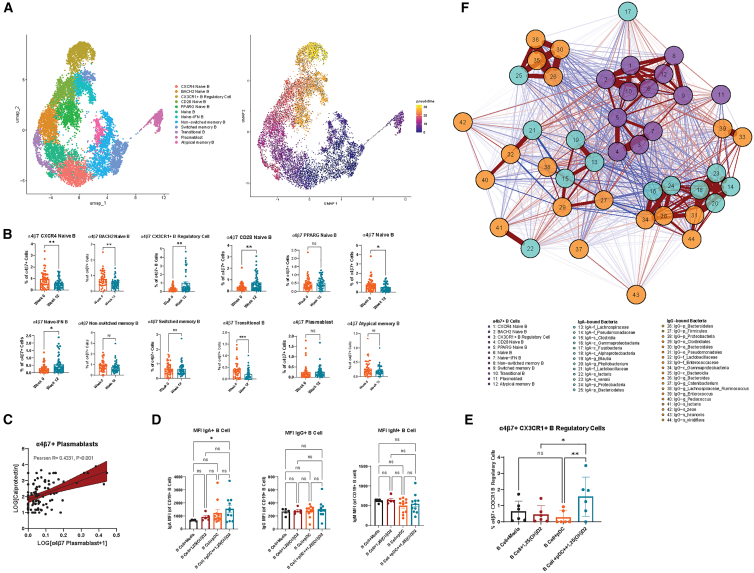
scRNA-seq reveals a4β7+ B cell heterogeneity and a4β7+ B cell immunophenotypes regulated by vitamin D (A and B) (A) UMAP of a4β7+ B cells (*n* = 48 patients, two time points) according to subtype (left) and pseudo time (right). (B) Differential abundance analyses of a4β7+ B cells reveals enrichment of a4β7+ CX3CR1 B regulatory cells with vitamin D. Data are represented as mean ± SEM. (C and D) (C) a4β7+ plasmablasts correlate with inflammation measured by fecal calprotectin. (D) Co-culture experiments of peripheral blood CD19^+^ B cells with pDCs (5,000 pDCs: 250,000 B cells) and 100 nM 1,25(OH)D2 for 48 h leads to increased IgA expression (measured my mean fluorescence intensity) but not IgG or IgM. Data are represented as mean ± SEM. (E) Co-culture experiments demonstrate that both pDCs and 1,25(OH)D2 together are needed to induce a4β7+ CX3CR1 B regulatory cells induction from peripheral blood CD19^+^ B cells. Data are represented as mean ± SEM. (F) Correlation network plot demonstrating association of a4β7+ B cells (purple nodes) with IgA- (turquoise nodes) and IgG-bound (orange nodes) gut microbiota taxa. Positive associations are denoted by red lines, and negative associations are denoted by blue lines. Weight of lines denote strength of correlations. Stars indicate nominal Wilcoxon signed-rank test *p* values: ns: *p* > 0.05; ∗*p* < 0.05; ∗∗*p* < 0.01; ∗∗∗*p* < 0.01.

Subclustering of the α4β7+ B cells (10.7K cells) yielded 12 transcriptionally distinct α4β7+ B cell subsets (Figure 5A, left). All α4β7+ B cells strongly expressed CD20 (MS4A1), except for the plasmablast cluster that strongly expressed CD319 (SLAM7). These subsets (key gene markers denoted in parentheses; Figure S11A) included CXCR4 naive B cell (*CD20*, *CXCR4*, *TCL1A*, *IGHM*, *IGHD*, and *JUND*), BACH2 naive B (*MS4A1*, *BACH2*, *SLC38A11*, and *ZNF630*), CX3CR1+ B regulatory cell (*MS4A1*, *CX3CR1*, *PRF1*, *GZMA*, *GZMB*, *GZMH*, *TGFBR3*, *PRDM1*, *IL10RA*, and *TGFBI*; Figure S11B), CD28 naive B (*MS4A1*, *CD28*, *LEF1*, and *INPP4B*), PPARG naive B (*MS4A1*, *PPARG*, *SMAD1*, and *IL1R1*), naive B (*MS4A1*, *IGHD*, *IGHM*, and *CD40*), naive-IFN B (*MS4A1*, *CCR4*, *IL32*, and *TNFRSF25*), non-switched memory B (*MS4A1*, *CD1C*, *TNFRSF13B*, and *GPR183*), switched memory B (*MS4A1*, *IGHE*, *ITGB1*, *CD27*, *CD82*, and *CD86*), transitional B (*MS4A1*, *IGHD*, *IGHM*, *MME (CD10)*, *CD24*, and *CD38*), plasmablast (*SLAM7/CD319*, *SDC1/CD138*, *CD38*, *IGHA1*, *IGHA2*, *IGHG1*, *IGHG2*, *IGHG3*, and *IGHG4*), and atypical memory B (*MS4A1*, *CD19*, *TBX21*, and *ITGAX*) cells. Trajectory analysis (Figure 5A, right) revealing pseudotime (number describing the relative position of a cell in the inferred developmental trajectory) of B cell subsets suggest that CXCR4 naive B cells have low pseudotime; plasmablasts, BACH2, and CD28 naive B cells have intermediate pseudotime; and CX3CR1+ B regulatory cells have the highest pseudotime (suggest furthest along differentiation/developmental pathway). In differential abundance analyses, vitamin D led to decreased α4β7+ CXCR4 B naive, BACH2 B naive, B naive, and transitional B cells and increased α4β7+ CX3CR1+ B regulatory cells, CD28 naive, and naive-IFN B cells (Figure 5B). Clinically, α4β7+ plasmablasts correlated positively with intestinal inflammation as measured by calprotectin (Pearson R = 0.4331, *p* < 0.001) (Figure 5C).

Given the increased B cell and pDC interactions from our scRNA-seq data, we next evaluated whether these cellular interactions and vitamin D were important in stimulating IgA expression in B cells and promoting B regulatory cell induction through co-culture experiments. The mean fluorescence intensity (MFI) of IgA+ B cells was significantly increased in the B cell+pDC+ vitamin D co-culture group compared to B cell only group, suggesting that both pDCs and vitamin D are needed to increase IgA+ B cells. There were no differences in MFI of IgG+ and IgM+ B cells with vitamin D and/or pDC co-cultures (Figure 5D). We also observed that B cell+pDC+ vitamin D co-culture group had increased percentage of α4β7+ CX3CR1+ B regulatory cells compared to B cell+ vitamin D or B cell+pDC alone. Our results suggest that vitamin D and pDCs may work synergistically to induce α4β7+ CX3CR1+ B regulatory cells from peripheral blood B cells (Figure 5E).

We performed correlation network analyses (Figures S12A and 5F; Table S6) to determine the association of α4β7+ B cells subsets with Ig-bound gut bacteria. α4β7+ CX3CR1+ B regulatory cells were positively associated with IgG-*p_Firmicutes* and negatively associated with IgA-*p_Proteobacteria*, IgG-*c_Gammaproteobacteria*, and IgG-*p_Proteobacteria*. α4β7+ plasmablasts were negatively associated with IgA-*s_veronii* and IgA-f*_Pseudomonadaceae*. α4β7+ CXCR4 naive B cells were positively associated with IgA*-f_Lactobacillaceae*, IgA-*c_Fusobacteria*, IgG-*f_Lactobacillaceae*, IgG-*f_Enterococcaceae*, and IgG-*g_Enterococcus*. α4β7+ BACH2 naive B cells were positively associated with IgG-*s_zeae*, IgG-*g_Lachnospiraceae_Ruminococcus* and IgA-*f_Lactobacillaceae*. α4β7+ transitional B cells were positively associated with IgA-*f_Lactobacillaceae*, IgG-*f_Enterococcaceae*, and IgG-*g_Enterococcus*.

### scRNA-seq reveals α4β7+ T cell heterogeneity and induction of α4β7+ T regulatory cells with vitamin D

Given the role of T cells in maintaining immune tolerance to gut microbiota (Gu et al., 2024) and their role in T-cell-dependent IgA induction in the gut (Benmark et al., 2012), we next examined the effects of vitamin D on α4β7+ T subsets and their association with Ig-bound gut bacteria. Our α4β7+ T cell scRNA-seq subset consisted of 36.7K CD4 T cells and 28.9K CD8 T cells.

**Figure 6:**
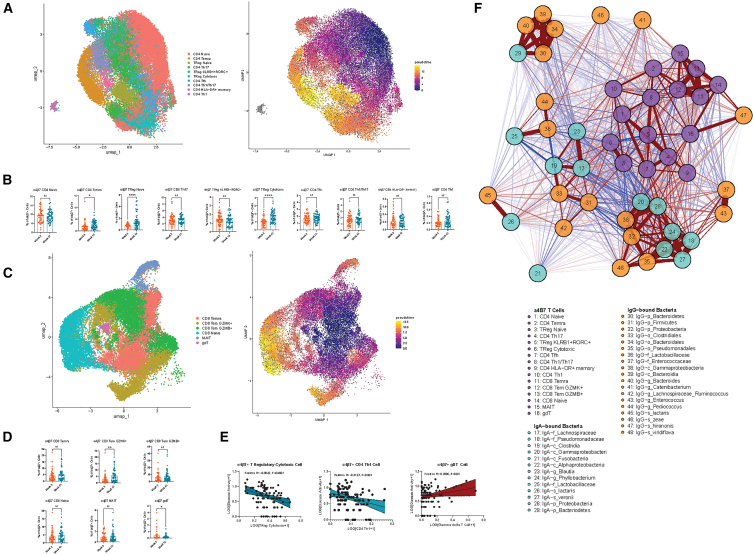
scRNA-seq reveals a4β7+ T cell heterogeneity and a4β7+ T cell immunophenotypes regulated by vitamin D (A and B) (A) UMAP of a4β7+ CD4 T cell subtypes (*n* = 48 patients, two time points) by cell type (left) and by pseudo time (right). (B) Differential abundance analysis reveals significant a4β7+ CD4 T cell subtypes altered by vitamin D including increased a4β7+ naive and cytotoxic T regulatory cells. Data are represented as mean ± SEM. (C and D) (C) UMAP of a4β7+ CD8 T cell subtypes by cell type (left) and by pseudo time (right). (D) Differential abundance analysis reveals significant a4β7+ CD8 T cell subtypes altered by vitamin D, including increased a4β7+ CD8 Tem GZMK+ and decreased a4β7+ gamma delta T cells. Data are represented as mean ± SEM. (E and F) (E) Linear regression analyses demonstrating specific a4β7+ T cells correlate with disease. (F) Correlation network plot demonstrating association of a4β7+ T cells (purple nodes) with IgA- (turquoise nodes) and IgG-bound (orange nodes) gut microbiota taxa. Positive associations are denoted by red lines, and negative associations are denoted by blue lines. Weight of lines denotes strength of correlations. Stars indicate nominal Wilcoxon signed-rank test *p* values: ns: *p* > 0.05; ∗*p* < 0.05; ∗∗*p* < 0.01; ∗∗∗*p* < 0.01.

We identified 10 transcriptionally distinct α4β7+ CD4 T cell subsets annotated using gene signatures (100 top genes) using a previously published PBMC scRNA-seq reference (Terekhova et al., 2023): CD4 naive, CD4 Temra, TReg naive, CD4 Th17, TReg KLRB1+RORC+, TReg cytotoxic, CD4 Tfh, CD4 Th1/Th17, CD4 HLA−DR + memory, and CD4 Th1 (Figure 6A, left). In trajectory analyses, α4β7+ CD4 naive cells had the lowest pseudotime; CD4 Th1 and CD4 Th1/Th17 cells had intermediate pseudotime; and CD4 Temra, TReg naive, and TReg cytotoxic cells had the highest pseudotime (Figure 6A, right). In differential abundance analysis, vitamin D led to increased α4β7+ CD4 Temra, TReg naive, and TReg cytotoxic cells (Figure 6B).

We identified six transcriptionally distinct α4β7+ CD8 T cell subsets: CD8 Temra, CD8 Tem GZMK+, CD8 Tem GZMB+, CD8 naive, MAIT, and gdT (Figure 6C, left). In trajectory analyses, α4β7+ CD8 Temra and CD8 Tem GZMB+ had the lowest pseudotime, whereas CD8 Tem GZMK+ had the highest (Figure 6C, right). In differential abundance analysis, vitamin D led to increased α4β7+ CD8 Tem GZMK+ cells and decreased α4β7+ gamma delta T cells (gdT) (Figure 6D). Clinically, α4β7+ TReg cytotoxic cells (Pearson R = −0.8022, *p* < 0.0001) and CD4 Th1 cells (Pearson R = −0.3123, *p* < 0.001) were inversely associated with disease activity scores. There was a trend toward positive correlation (Pearson R = 0.2006, *p* = 0.05) between α4β7+ gdT cells and disease activity (Figure 6E).

In correlation network analyses (Figures S12B and 6F; Table S7), α4β7+ TReg naive and α4β7+ TReg cytotoxic cells were positively associated with IgG-*o_Clostridiales*, IgG-*p_Firmicutes*, and IgA-*f_Lachnospiraceae* and negatively associated with IgA-*p_Proteobacteria* and IgG-*p_Proteobacteria*. α4β7+ CD4 Temra were positively associated with IgG-*g_Pediococcus*. α4β7+ CD4 Th17 were positively associated with IgA*-g_Phyllobacterium*, IgA-*c_Alphaproteobacteria*, IgA-*s_veronii*, IgA-*f_Pseudomonadaceae*, IgG-*g_Enterococcus*, and IgG-*f_Enterococcaceae* and negatively associated with IgG-*f_Lactobacillaceae*. α4β7+ gdT cells were positively associated with IgG-*s_hiranonis*, IgA-*p_Proteobacteria*, IgA-*c_Gammaproteobacteria*, IgG-*c_Gammaproteobacteria*, and IgG-*p_Proteobacteria*. α4β7+ CD8 Tem GZMB+ cells were positively associated with IgG-*s_hiranonis*.

## Discussion

Low vitamin D status in patients with IBD is associated with increased risk of clinical relapse,^33^ disease activity, and intestinal inflammation.^34^^,^^35^ Here, we report that a 12-week vitamin D intervention in patients with IBD and low vitamin D was associated with improvements in disease activity and fecal calprotectin, with concurrent alterations in host immune-microbe interactions via IgA and IgG binding to specific bacterial taxa and α4β7+ peripheral blood immunophenotypes. While prior cross-sectional studies have characterized the composition of IgA-^14^ and IgG-bound^17^ gut bacteria in patients with IBD and their associations with therapy and clinical outcomes, our prospective multi-omics study demonstrates the ability to reprogram Ig binding to gut microbiota and immune tolerance with a specific nutritional intervention.

Mucosal IgA plays a critical role in intestinal immune homeostasis. IgA deficiency can lead to spontaneous inflammation in the gastrointestinal tract,^36^ enrichment of proinflammatory gut bacteria,^37^ and increased gut microbiota systemic immune response.^38^ IgA-bound gut bacteria such as *Odoribacter splanchnicus* have been associated with efficacy of fecal microbial transplant (FMT) in patients with UC and can directly limit colitis.^39^ Therefore, increasing endogenous IgA binding to gut microbiota could have protective and anti-inflammatory effects. Our prospective study identified vitamin D treatment as a strategy to increase IgA-bound gut microbiota in patients with IBD and select for IgA binding to several beneficial bacterial taxa (*f_Lachnospiraceae*, *g_Blautia)*. *Blautia* can stimulate colonic mucus growth and intestinal barrier integrity through the production of the short-chain fatty acids (SCFA).^40^ *Lachnospiraceae* are SCFA producers^41^ and known to convert primary bile acids by 7α-dehydroxylation to secondary bile acids,^42^ which have anti-inflammatory effects on IBD.^43^ Our study also revealed that vitamin D increased the proportion of α4β7+ T regulatory cells. Our findings are consistent with prior studies revealing a role for vitamin D in inducing differentiation of T regulatory cells^44^^,^^45^ and expand these observations to also include gut tropic α4β7+ T regulatory cells. Interestingly, IgA-f_*Lachnospiraceae* (flagellated bacteria) were positively associated with α4β7+ T regulatory cells in our study. This parallels prior studies demonstrating that T regulatory cells regulate IgA responses to microbial antigens such as flagellin^46^ through a symbiotic regulatory loop.^47^ Taken together, our data suggest that vitamin D may increase IgA binding to gut microbiota by inducing T regulatory cells and have additional anti-inflammatory effects through selection of IgA-bound gut bacteria that produce SCFA and secondary bile acids.

While IgA binding to gut microbiota is protective and homeostatic, IgG binding to gut bacteria is considered proinflammatory and triggers type 17 inflammation in ulcerative colitis through engagement of Fcγ receptors on phagocytes.^16^ In our study, IgG-bound gut bacteria, mostly IgG-*p_Proteobacteria* and IgG-*p_Bacteroidetes*, positively correlated with disease activity and markers of inflammation (CRP, fecal calprotectin). We also found that *IgG-f_Enterococcaceae* were associated with proinflammatory α4β7+ CD4 Th17 cells. Our vitamin D intervention decreased overall IgG binding to gut microbiota with most taxa belonging to p_*Proteobacteria and* f_*Enterococcaceae*. *Proteobacteria*, which include *Escherichia coli* with adherent-invasive capabilities, are considered proinflammatory and play a pathogenic role in IBD.^48^ Increased antibodies targeting a subset of Proteobacteria (e.g., *E*. *coli* antigens) have been associated with severe phenotypes, frequent disease progression, longer disease duration, and a greater need for surgery in patients with Crohn's disease.^49^ *Enterococcaceae* are increased in patients with IBD^50^^,^^51^ and can trigger intestinal inflammation, dysplasia, and carcinoma in mice models of IBD.^52^ While our study demonstrated that vitamin D can reduce IgG-p_*Proteobacteria* and IgG-f_*Enterococcaceae* in patients with IBD, further work is needed to determine how reductions in these specific host-immune microbe interactions may alter IBD phenotypes and clinical outcomes. The mechanisms that regulate isotype switching from homeostatic IgA to proinflammatory IgG in intestinal inflammation are poorly understood. Our BCR repertoire isotype usage analyses revealed that vitamin D increased BCR isotype usage of IgA1 but had no significant effects on IgG isotype usage. Thus, our data may suggest that the decreased IgG binding to gut bacteria with vitamin D may be an indirect effect of increased IgA-gut microbe binding, which may compete with IgG in binding to bacterial epitopes rather than directly regulating IgG isotype switching.

To infer potential mechanisms of increased IgA binding to gut microbiota with vitamin D, we performed cell-cell interaction and systems signaling pathway analyses using scRNA-seq. Our study demonstrated that vitamin D increased BAFF signaling between pDCs and B cell subsets. Interestingly, a prior study demonstrated that pDC co-culture with naive B cells induced IgA isotype switching, which was dependent on BAFF expression by pDCs.^53^ To clarify the role of vitamin D in inducing IgA+ B cells by pDCs, we performed co-culture experiments with B cells and pDCs. We found that both the active form of vitamin D (1,25(OH)D2) and pDCs were needed to increase IgA+ CD19^+^ B cells. Taken together, our data suggest that vitamin D may also increase IgA binding to gut microbiota through induction of IgA+ B cells via increased pDC-B cell interactions and BAFF signaling.

We investigated the immune landscape of peripheral gut tropic α4β7+ B cells using scRNA-seq. Notably, we found that vitamin D increased the proportion of α4β7+ CX3CR1 B regulatory cells (while decreasing immature α4β7+ naive B cells) that have increased expression of cytotoxic genes (*PRF1*, *GZMA*, *GZMB*, *GZMH*), *TGFBR3*, and *IL10RA*). Tolerogenic B regulatory cells have been shown to attenuate chronic inflammation in IBD^54^ and that CX3CR1 B regulatory cells can effectively suppress effector CD4 T cell activation.^55^ To clarify whether vitamin D can directly induce differentiation of peripheral B cells into α4β7+ CX3CR1 B regulatory cells, we performed co-culture experiments with vitamin D, B cells, and pDCs. We found that the presence of both pDCs and 1,25(OH)D2 was necessary to induce α4β7+ CX3CR1 B regulatory cell differentiation *in vitro*. Prior work has revealed that the gut microbiome and their metabolites support B regulatory cell development and function in the gastrointestinal tract.^56^^,^^57^ To infer B regulatory cell-gut microbiome interactions, we performed correlation analyses, which revealed that α4β7+ CX3CR1 B regulatory cells were positively associated with IgG-p_*Firmicutes*, which are known SCFA producers. Although the functional significance of this interaction is unclear, a prior study demonstrated that microbiota-derived SCFA butyrate supports the function of B regulatory cells via activation of the aryl-hydrocarbon receptor.^57^ Future work is warranted to explore the mechanisms of B regulatory cell-gut microbiome interactions and therapeutic potential of vitamin-D-induced α4β7+ CX3CR1 B regulatory cells in patients with IBD.

In summary, our study demonstrated that vitamin D could promote immune tolerance to commensal gut microbiota in patients with IBD through increased IgA and decreased IgG binding to gut bacteria. Specifically, our study demonstrated vitamin D enrichment of IgA-bound beneficial bacteria (with known SCFA and secondary-bile-acid-producing phenotypes) and decrease in IgG-bound proinflammatory bacteria previously implicated in the pathogenesis of IBD. In addition, our study revealed that vitamin D regulates tolerogenic pathways including BAFF signaling between pDCs and B cells and through induction of anti-inflammatory α4β7+ B and T regulatory cells. Our study also generated prospective immune repertoire datasets and identified BCR and TCR clonotypes that are associated with specific Ig-bound gut bacterial taxa. Our study highlights the malleability of the immune repertoire and the ability to reprogram BCR and TCR clonotypes through a dietary intervention such as vitamin D supplementation. Overall, our results reveal a strategy to regulate immune tolerance to gut microbiota in IBD and provides a foundation for manipulating host immune-microbe interactions to boost immune tolerance and address a critical component in the pathogenesis of IBD.

### Limitations of the study

Some caution is warranted in interpreting our results due to inherent limitations. While we performed a prospective study of vitamin D treatment of a well-phenotyped cohort of patients with IBD, our vitamin D intervention was not randomized or placebo-controlled. Instead, vitamin D treatment was compared against low vitamin D status baseline for the same group of patients, which avoids inter-person heterogeneity in baseline gut microbiome composition and immune repertoires. Despite this, external confounding effects were minimized, as there were no medication changes or new antibiotic exposures during the vitamin D course. In addition, we included *in vitro* co-culture studies with vitamin D (which included vitamin D placebo controls), which complemented and supported our clinical observations in patients with IBD. In addition, our study did not take into account vitamin D derived from dietary sources or sun exposure. Further studies are needed to evaluate whether raising vitamin D levels through diet and/or sun exposure recapitulates the effects of oral vitamin D supplementation. We also only assessed two time points with vitamin D, thus the effect size and durability of our findings beyond 12 weeks remain unclear. Finally, our study used 16S rRNA sequencing instead of metagenomic sequencing and cannot consider effects on viruses and fungi within the gut microbiome community or metagenome function. Despite these inherent limitations, our study reveals mechanistic insights into regulation of immune tolerance to gut microbiota and immune repertoire-gut microbiome relationships in IBD through a specific nutritional intervention such as vitamin D supplementation. Given the scarcity of high-quality prospective human host immune-gut microbiome studies, our study has important clinical implications and can inform future studies aimed at personalized nutrition to regulate immune responses in patients with chronic inflammatory and autoimmune conditions.

## Resource availability

### Lead contact

Further information and requests for resources and reagents should be directed to and will be fulfilled by the lead contact, John Gubatan (gubatan.johnmark@mayo.edu).

### Materials availability

This study did not generate new unique reagents.

### Data and code availability

• All data are available upon request to the lead contact, John Gubatan (gubatan.johnmark@mayo.edu). All scRNA-seq and immune repertoire data generated during this study are available at the Gene Expression Omnibus (GEO) under accession number GSE319270. All IgA-SEQ and IgG-SEQ data generated under this study are available at Gene Expression Omnibus (GEO) under accession number GSE319142. All whole-stool 16S data generated under this study are available at Gene Expression Omnibus (GEO) under accession number GSE319268.
• No custom code was generated in this study.
• Any additional information required to reanalyze the data reported in this work paper is available from the lead contact upon request.

## Acknowledgments

We wish to thank the patient participants for their engagement and effort to enable this study. J.G. and this project were supported in part by a Doris Duke Physician Scientist Fellowship Award (grant no. #2021091), CZ Biohub Physician Scientist Scholar Award, 10.13039/100000002NIH 10.13039/100000062NIDDK LRP Award (2L30 DK126220), 10.13039/100020710Stanford Translational Research and Applied Medicine (TRAM) Scholar Award, and Stanford 10.13039/100015521MCHRI Pediatric IBD and Celiac Disease Research Award. We thank Helen Smith, PhD from BD Biosciences for technical support with the BD Rhapsody single-cell platform.

## Author contributions

J.G. conceived and designed the study, obtained funding, and wrote the manuscript. R.S., J.H., T.F., and M.T. recruited patients, obtained and processed blood and stool samples, and maintained clinical trial metadata at Stanford site. T.B. and O.H.N. led patient recruitment, sample collection, and processing at Denmark site. S.R. and S.R.S. provided laboratory and clinical research coordinator support. R.S., J.Y., and J.H. performed bacterial FACS and Ig-seq experiments. J.G., R.S., J.Y., and J.H. performed scRNA-seq experiments. S.S. and J.S. provided guidance on gut microbiome experiments, data analyses, and interpretation. J.G. performed gut microbiome analyses. J.G. performed scRNA-seq and immune repertoire analyses. J.G. created manuscript figures and tables. P.K. assisted with BCR metric data processing and analyses. Y.R., P.K., and S.B. provided feedback on immune repertoire and B cell analyses and data interpretation. S.R., M.J.R., O.H.N., S.B., J.S., and S.R.S. provided critical feedback on data presentation and manuscript.

## Declaration of interests

10.13039/100005564The authors declare no competing interests.

## STAR★Methods

### Key resources table

**Table:** 

REAGENT or RESOURCE	SOURCE	IDENTIFIER
**Antibodies**		

Anti-Human IgA-APC	REAfinity™	Catalog No. 130-116-879; RRID:AB_2727810
Anti-Human IgG-PE	REAfinity™	Catalog No. 130-119-878; RRID:AB_1036187
Anti-APC MicroBeads	Miltenyi Biotec	Catalog No. 130-090-855; RRID:AB_244367
Anti-PE MicroBeads	Miltenyi Biotec	Catalog No. 130-048-801; RRID:AB_244373
F(ab')_2_ Anti-Human IgM	Jackson ImmunoResearch	Cat. No.109-006-129; RRID:AB_2337680
anti-CD19	BD Biosciences	Cat. No.560353; RRID:AB_1645564
anti-CD27	BD Biosciences	Cat. No. 771531; RRID:AB_3693363
anti-CD38	BD Biosciences	Cat. No. 555462; RRID:AB_398599
anti-IgD	BD Biosciences	Cat. No. 770037; RRID:AB_3691907
anti-IgM	BD Biosciences	Cat No. 555750; RRID:AB_396092
anti-IgG	BD Biosciences	Catalog No. 563246; RRID:AB_2738092
anti-α4β7	R&D systems	Cat. No. FAB10078R; RRID:AB_3644725
anti-CX3CR1	BioLegend	Cat. No. 341629; RRID: AB_2814256
anti-CD304	BD Biosciences	Cat. No 749092; RRID: AB_2873484

**Biological samples**		

Peripheral blood mononuclear cells	Stanford University and Universtiy of Copenhagen IBD Centers	Stanford University (IRB 60958), University of Copenhagen (IRB 2021-1696)
Stool	Stanford University and Universtiy of Copenhagen IBD Centers	Stanford University (IRB 60958), University of Copenhagen (IRB 2021-1696)

**Chemicals, peptides, and recombinant proteins**		

PBS - Phosphate-Buffered Saline (10×) pH 7.4, RNase-free	Invitrogen	Catalog No. AM9624
N-acetyl-cysteine	Thermo Scientific Chemicals	Catalog No. 616-91-1
Ficoll	GE Healthcare	Catalog No. 14-1440-03)
Gibco Recovery™ Cell Culture Freezing Medium	Gibco	Catalog No. 12648010
Penicillin	ThermoFisher	Catalog No. 15140-122
Streptomycin	ThermoFisher	Catalog No. 15140-122
Gentamicin	ThermoFisher	Catalog No. 15750-060
1,25(OH)D2	Sigma Aldrich	Catalog No. 740578

**Critical commercial assays**		

Serum 25(OH)D assay on DXI600	Beckman Coulter	Catalog No. B46328
Serum C-reactive protein (CRP) Immunoassay	Roche Diagnostics	Catalog No. 07876033190
Fecal calprotectin ELISA kit	Buhlmann	Catalog No. 200096
IgA ELISA Kit	Invitrogen	Catalog No. BMS2096
IgG ELISA Kit	Invitrogen	Catalog No. BMS2091
Microbiome DNA Purification Kit	PureLink™	Catalog No. A29790
Accuspin tubes	Sigma-Aldrich	Catalog No. A2055
BD® Human Single-Cell Multiplexing Kit	BD Biosciences	Catalog No. 633781
BD Rhapsody™ Cartridge	BD Biosciences	Catalog No. 633733
BD Rhapsody™ TCR/BCR Amplification Kit	BD Biosciences	Catalog No. 665345
BD Rhapsody™ WTA Amplification Kit	BD Biosciences	Catalog No. 633801
Miltenyi B Cell Isolation Kit II, human	Miltenyi	Catalog no. 130-091-151)
Miltenyi CD304 BDCA-4/Neuropilin-1 MicroBead Kit, human	Miltenyi	Catalog no. 130-090-532

**Deposited data**		

scRNA-seq and immune repertoire data	Gene Expression Omnibus (GEO), https://www.ncbi.nlm.nih.gov/geo/	GSE319270: https://www.ncbi.nlm.nih.gov/geo/query/acc.cgi?acc=GSE319270
IgA-SEQ and IgG-SEQ data	Gene Expression Omnibus (GEO), https://www.ncbi.nlm.nih.gov/geo/	GSE319142: https://www.ncbi.nlm.nih.gov/geo/query/acc.cgi?acc=GSE319142
Whole stool 16 S data	Gene Expression Omnibus (GEO), https://www.ncbi.nlm.nih.gov/geo/	GSE319268: https://www.ncbi.nlm.nih.gov/geo/query/acc.cgi?acc=GSE319268

**Experimental models: Cell lines**		

B cell primary cells	Extracted from peripheral blood mononuclear cells from IBD patient cohort from study	N/A
Plasmacytoid dendritic cells primary cells	Extracted from peripheral blood mononuclear cells from IBD patient cohort from study	N/A

**Oligonucleotides**		

Bacterial 16 S sequencing of the V4 region	Novogene	primer 515F/sequence GTGCCAGCMGCCGCGGTAA
Bacterial 16 S sequencing of the V5 region	Novogene	primer 907R/sequence CCGTCAATTCCTTTGAGTTT

**Software and algorithms**		

BD Rhapsody™ Sequence Analysis Pipeline	BD Rhapsody™	Revision 15
R Statistical Software	R Core Team	RRID:SCR_001905, https://www.r-project.org/
Seurat v5 (R package)	Satija Lab	RRID:SCR_007322, https://satijalab.org/seurat/
Harmony (R Package)	https://github.com/immunogenomics/harmony	immunogenomics/harmony (GitHub)
Quantitative Insights into Microbial Ecology (QIIME) 2	http://qiime.org	Version 2023.9
DADA2	https://github.com/benjjneb/dada2	N/A
microbiomeMarker (R package)	https://github.com/yiluheihei/microbiomeMarker	N/A
PICRUSt2 (Phylogenetic Investigation of Communities by Reconstruction of Unobserved States)	https://huttenhower.sph.harvard.edu/picrust/	N/A
Monocle3 (R package)	https://cole-trapnell-lab.github.io/monocle3/	N/A
CellChat v2 (R package)	https://github.com/jinworks/CellChat	N/A
Immunarch v0.9 (R package)	https://immunarch.com/	N/A
SHhazaM v1.3.1 (R package)	https://github.com/immcantation/shazam	N/A
EnhancedVolcano v1 (R package)	https://github.com/kevinblighe/EnhancedVolcano	N/A
qgraphh v1.9.8 (R package)	https://github.com/cran/qgraph	N/A
GraphPad Prism	GraphPad	v10
phyloseq v4.5 (R package)	https://joey711.github.io/phyloseq/	N/A

### Experimental model and study participant details

#### Vitamin D inflammatory bowel disease clinical trial NCT04828031

Patients with established inflammatory bowel disease (ulcerative colitis or Crohn’s disease) were screened and invited to participate in vitamin D prospective study at Stanford University (IRB 60958) and the University of Copenhagen (IRB 2021-1696). Patients who met inclusion criteria (adult patients (18 years or older) with inflammatory bowel disease (ulcerative colitis or Crohn’s disease), low serum vitamin D (25(OH)D ≤ 25 ng/mL, not on vitamin D supplementation at time of recruitment, no prior bowel resections, no antibiotic use in past 3 months) were enrolled. Patients who had prior bowel surgeries (colectomy, small bowel resections), renal dysfunction, history of hypercalcemia, history of HIV (human immunodeficiency virus), history of IgA deficiency, history of common variable immunodeficiency (CVID), or current or recent C. diff infection were excluded from study. All patients provided consent in accordance with Institutional Review Board guidance for each institution as noted above. Patient characteristics are shown in Table 1. At the time of enrollment (week 0), patients had blood and stool samples collected. Patients were then treated with 50,000 units of oral vitamin D (ergocalciferol) once per week for 12 weeks. Blood and stool samples were collected at the end of study (week 12). C-reactive protein and fecal calprotectin were measured from week 0 and week 12 blood and stool samples, respectively. Disease activity scores (partial Mayo score for ulcerative colitis, Harvey Bradshaw Index for Crohn’s disease), quality of life scores (short inflammatory bowel disease questionnaire/SIBDQ) were collected at week 0 and week 12. Forty-eight patients with inflammatory bowel disease (IBD) were included in this analysis. The average age of patients was 38.96 years. About 45.8% of patients were men and 54.2% were woman (clinical metadata summarized in Table S8). Clinical trial is registered under NCT04828031.

### Method details

#### Vitamin D, CRP, and fecal calprotectin measurements

Serum vitamin D (25-hydroxyvitaminD/25(OH)D) was measure using a two-site immunoenzymatic “sandwich” assay on DXI600 (Beckman Coulter, Catalog No. B46328). Serum C-reactive protein (CRP) was quantified using a particle enhanced turbidimetric inhibition immunoassay (Roche Diagnostics, Catalog No. 07876033190). Fecal calprotectin extraction was performed using the Buhlmann Calprotectin ELISA kit (Catalog No. 200096) according to the manufacturer’s instructions (Alpco Immunoassays).

#### Stool processing and bacterial fluorescence-activated cell sorting (FACS)

Frozen stool (200 mg) samples from each patient were thawed and dissolved in 1 mL of phosphate buffered saline (Invitrogen, Catalog No. Catalog No. AM9624). Stool was resuspended in 1 mL of 5 mM N-acetylcysteine (Thermo Scientific Chemicals, Catalog No. 616-91-1) to break disulfide bonds in mucus and release bacterial cells and then passed through 100 μM filters and centrifuged. The resulting stool pellets were resuspended in 1 mL of PBS and divided into four fractions for downstream experiments (bacterial FACS, whole microbiome 16 S, IgA-Seq, and IgG-Seq). For bacterial FACS, stool cell pellets were centrifuged and resuspended in PBS and stained with 1:10,000 SYBR Green (Sigma Aldrich) at room temperature in dark for 20 min. Cell pellets were subsequently centrifuged, resuspended in PBS, and stained with 1:250 Anti-Human IgA-APC (REAfinity, Catalog No. 130-116-879) and 1:250 of Anti-Human IgG-PE (REAfinity, Catalog No. 130-119-878) and incubated on ice for 20 min. Cell pellets were centrifuged and resuspended in FACS buffer and 1:50 Fc block was to each sample and incubated at room temperature for 10 min. Bacterial cell pellets were then centrifuged and resuspended in FACS buffer. FACS was used to quantify the proportion of SYBR green positive (live bacterial cells) that were IgA-APC+, IgG-PE+, and IgA+IgG + double-positives.

#### Measurement of stool and serum immunoglobulins

Stool supernatants from frozen stool resuspended in PBS were diluted 1:100 and used to measure secretory IgA and IgG levels via ELISA kits according to manufacturer’s instructions (Invitrogen, Catalog No. BMS2096 for IgA, Catalog No. BMS2091 for IgG). Likewise, serum samples from patients were used to measure serum IgA and IgG levels using the same ELISA kits (Invitrogen, Catalog No. BMS2096 for IgA, Catalog No. BMS2091 for IgG).

#### IgA-Seq, IgG-Seq, and whole gut microbiome 16 S sequencing and processing

Stool pellets from each patient were processed as previously described. Bacterial cell pellets were resuspended in PBS and separate stool fractions were stained with either 1:250 Anti-Human IgA-APC (REAfinity, Catalog No. 130-116-879) or 1:250 of Anti-Human IgG-PE (REAfinity, Catalog No. 130-119-878) and incubated on ice for 20 min. Bacterial cell pellets were centrifuged and then washed with MACS buffer. Anti-PE MicroBeads (Miltenyi Biotec, Catalog No. 130-090-855) were added to IgG fraction and Anti-APC MicroBeads (Miltenyi Biotec, Catalog No. 130-048-801) to IgA fraction. Bacterial cell pellets were then centrifuged, resuspended in MACS buffer, and placed onto OctoMACS Separator (Miltenyi Biotec, Catalog No. 130-042-108) columns for magnetic separation. IgA and IgG-bound bacterial fractions were then collected for each sample. Purity of IgA-bound and IgG-bound gut bacteria were above 90% for all samples included in downstream analyses. DNA from bacterial cell pellets from IgA-bound, IgG-bound, and unfractionated (whole gut microbiome) samples were isolated using the PureLink Microbiome DNA Purification Kit (Catalog No. A29790) per manufacturer’s instructions. Bacterial 16 S sequencing of the V4 and V5 regions (primer 515F/sequence GTGCCAGCMGCCGCGGTAA, primer 907R/sequence CCGTCAATTCCTTTGAGTTT) were performed at Novogene using 2 × 250 sequencing on an Illumina MiSeq.

#### Peripheral blood mononuclear cell (PBMC) scRNA-seq, scBCR-seq, scTCR-seq processing

Peripheral blood mononuclear cells (PBMC) were isolated from whole blood. In brief, blood samples (5–10 mL) were poured into Accuspin tubes (Sigma-Aldrich, Catalog No. A2055) layered with Ficoll (GE Healthcare, Catalog No. 14-1440-03) and centrifuged to generate a buffy coat layer. PBMCs were isolated from the buffy coat layer, concentrated, and placed in Gibco Recovery Cell Culture Freezing Medium (Catalog No. 12648010). Samples were kept in Mr. Frosty containers in −80C freezer and subsequently stored in liquid nitrogen until downstream experiments. The BD Human Single-Cell Multiplexing Kit (Catalog No. 633781) was used to multiplex PBMCs from each patient in batches of 12 patients. About 5,000–10,000 multiplexed PBMCs from each patient were loaded into the BD Rhapsody Cartridge (BD Biosciences, Catalog No. 633733). Single-cell capture, barcoding, lysis, and cDNA synthesis were performed with the BD Rhapsody Express Single-Cell Analysis System according to the manufacturer’s instructions. Paired TCR/BCR Full Length (BD Rhapsody TCR/BCR Amplification Kit, Catalog No. 665345) and mRNA Whole Transcriptome Analysis (WTA) (BD Rhapsody WTA Amplification Kit, Catalog No. 633801) libraries were indexed according to manufacturer’s instructions. Libraries were sequenced using the NovaSeq X Plus sequencer at a depth of 25,000 reads for WTA and 5,000 reads per cell for TCR and BCR. Fastq files were processed using BD’s Rhapsody analysis pipeline on the Seven Bridges Platform using default parameters and the human reference genome, GRCh38-PhiX-gencodev29 to generate raw cell by gene matrices. The R package Seurat V5^58^ was used to filter out low quality cells (less than 200 genes or more than 20,000 RNA counts per cell) and perform standard scRNA-seq processing including gene count log normalization, scaling, dimensionality reduction, principal component analyses (PCA), and generation of Uniform Manifold Approximation and Projection (UMAP) reduction. To reduce technical and biological batch variability, all multiplexed sample sets were merged and then integrated using the Harmony package.^59^

#### B cell and plasmacytoid dendritic cell co-culture vitamin D experiments

PBMC samples obtained before vitamin D treatment from 6 to 12 IBD patients from our clinical trial cohort (vitamin D < 20 ng/mL, all inflamed patients based on calprotectin) were used to isolate peripheral B and plasmacytoid dendritic cells for co-culture experiments. B cells were isolated using a negative selection MACS separation kit (Miltenyi B Cell Isolation Kit II, human, Catalog no. 130-091-151) according to manufacturer’s instructions. Plasmacytoid dendritic cells (pDCs) were isolated from PBMC using a positive selection MACS separation kit (Miltenyi CD304 BDCA-4/Neuropilin-1 MicroBead Kit, human, Catalog No. 130-090-532). Peripheral blood CD19^+^ B cells were co-cultured with pDC (5000 pDC: 250,000 B cell ratio) or without pDC in 6 well plates (culture media RPMI 1640 + 100 U/mL penicillin (ThermoFisher, Catalog No. 15140-122), 100 mg/mL streptomycin (ThermoFisher, Catalog No. 15140-122)+ 50 mg/mL gentamicin (ThermoFisher, Catalog No. 15750-060])+ FBS 2% with 100 nM of 1,25(OH)D2 (Sigma Aldrich, Catalog No. 740578) or vehicle (EtOH+ media) control for 48 h. F(ab')_2_ Anti-Human IgM (10 μg/mL) (Jackson ImmunoResearch, Cat. No.109-006-129) was added to culture media for BCR stimulation. Cells were then isolated and stained with anti-CD19 (BD Biosciences, Cat. No.560353), anti-CD27 (BD Biosciences, Cat. No. 771531), anti-CD38 (BD Biosciences, Cat. No. 555462), anti-IgD (BD Biosciences, Cat. No. 770037), anti-IgM (BD Biosciences, Cat No. 555750), anti-IgA (Miltenyi, Cat. No. 130-113-475), anti-IgG (BD Biosciences, Catalog No. 563246), anti-α4β7 (R&D systems, Cat. No. FAB10078R), anti-CX3CR1 (BioLegend, Cat. No. 341629), and anti-CD304 (BD Biosciences, Cat. No 749092) for FACS analyses. Mean fluorescence intensity (MFI) of IgA, IgG, and IgM in CD19^+^ B cells and percentage of α4β7+ CX3CR1+ B regulatory cells in each experimental condition were quantified using FACS.

#### Microbiome analyses

Analyses of 16 S rRNA data were performed using the Quantitative Insights into Microbial Ecology (QIIME) 2 (http://qiime.org), an open-source bioinformatics pipeline for performing analysis of microbiome sequence data as previously described.^60^ Briefly, raw sequencing data were demultiplexed using unique barcodes assigned to each sample and then denoised using Dada2 as previously described.^61^ Remaining reads were then clustered into Operational Taxonomic Units (OTUs) using Greengenes v13-8 as a reference set to assign taxonomy to each OTU.^62^ OTU tables were rarified at the sequencing depth of 1,000 sequences/sample. Alpha diversity (bacterial richness of a sample expressed as a function of the number of OTUs identified in it) was estimated using Shannon and Chao1 diversity.^63^ Beta diversity (distance between samples based on differences in OTUs present in each sample) was measured using Bray curtis dissimilarity index^64^ calculated from the rarefied OTU tables. Principal coordinate analysis (PCoA) was used to visualize clustering patterns between samples based on beta diversity distances. Association between microbiome composition and covariates were tested using PERMANOVA via adonis2, a nonparametric test similar to ANOVA but that does not require the data to be normally distributed.^65^ Significance of PERMANOVA tests were determined using 999 permutations with adjustment for multiple testing. Linear discriminant analysis effect size (LEfSe) was used to identify bacterial taxa differentially enriched in IgA- or IgG-bound gut bacteria before and after vitamin D treatment.^66^ The R package microbiomeMarker^67^ was used to generate barplots and cladograms from Lefse analyses. PICRUSt2 (Phylogenetic Investigation of Communities by Reconstruction of Unobserved States) was used to infer functional differences in metabolic pathways for Kyoto Encyclopedia of Genes and Genomes (KEGG) among IgA- and IgG-bound gut bacteria with vitamin D treatment.^68^ We validated the LEFSe results by calculating the abundance of IgA-binding and IgG-binding to specific bacteria taxa using the Palm Index^15^ and IgA probability ratio and IgG probability ratio (extrapolated from IgA methods) as previously described.^69^

#### scRNA-seq cell annotation and subclustering

The merged PBMC single-cell dataset (375 K cells) was annotated using a reference-mapping approach with Seurat using a previously published PBMC single-cell atlas.^70^ In brief, the merged PBMC Seurat object was normalized with SCTransform() to match the PBMC reference. Anchors then were found between query cells and reference using a precomputed PCA (spca) transformation. Cell type labels were then transferred from the reference to query and project query data onto the UMAP structure of the reference. To focus on α4β7+ immune cells, cells co-expressing the integrin genes at ITGA4 >0.5 & ITGB7 >0.5 expression levels were filtered using the Seurat subset function and labeled as α4β7+ cells. To further investigate α4β7+ B and T cell heterogeneity, B cell, CD4 T cell, and CD8 T cell clusters were filtered from the PBMC dataset and further subclustered using a higher resolution (2) with Seurat. The R package scType^71^ was used to annotate the B, CD4, and CD8 T cell subclusters using a previously published single-cell dataset with deeper B and T cell subcluster annotations.^72^ As sctype generated several α4β7+ B naive and switched memory B cell subclusters, these subclusters were further manually annotated using highly expressed gene markers with known B cell function from the FindAllMarkers Seurat function.

#### Differential abundance and trajectory analyses

We performed differential abundance analysis to compare the number of immune cells before (Week 0) and after (Week 12) vitamin D. Differential abundance calculates the total number of cells per condition at the per-sample level and tests the null hypothesis that the mean abundance between week 0 and week 12 samples equals zero. Cell abundance (proportion) was calculated by dividing the number of cells for a given type by total cells in each patient sample. Wilcoxon matched-pairs signed rank test was used to test the difference in cell type proportion between week 0 and week 12 samples. We performed trajectory analysis using Monocle 3^73^ to identify differences in α4β7+ B and T cell subset states. We first created a Seurat object that contained only α4β7+ B cells, α4β7+ CD4 T cells, α4β7+ CD8 T cells, which was reharmonized and reclustered to generate new UMAP dimension reduction values. We then converted our Seurat objecyts to the native Monocle 3 data format and estimated size factors. Next, we clustered the cells using the Leiden clustering algorithm with a k value 30 using Monocle3’s cluster_cells function. Additionally, we identified principal graphs from the reduced dimension space using reversed graph embedding with the learn_graph function. To identify root nodes for the origin of our pseudotime trajectories in an unbiased fashion, we used Monocle3’s get_earlierst_principal_node function when ordering cells. This assigned a pseudotime trajectory to all cells within our α4β7+ immune cell lineage, which we then used for downstream analysis. We generated a plot showing the expression of genes as a function of pseudotime using the plot_genes_in_pseudotime function within Monocle 3, filtering out for a minimum expression value of 0.5 for each of these genes to remove lowly expressing cells. We used the default formula, which uses a natural cubic spline with a degree of freedom of 3 using the ns function. The R package Qgraph^74^ was used to generate correlation network plots depicting association of α4β7+ B cells, α4β7+ CD4 T cells, α4β7+ CD8 T cells with Ig-bound gut bacteria. Qgraph^74^ was used to generate correlation network plots depicting association of α4β7+ B cells and T cells with Ig-bound gut bacteria.

#### Cell-cell interaction and systems signaling pathway analyses

Cell-cell interaction, systems signaling, and ligand-receptor analyses were performed using CellChat v2.^75^ We created subsets of our PBMC Seurat object according to vitamin D status (week 0, week 12). We then created CellChat objects for vitamin D week 0 and week 12 samples and merged them together into one CellChat object for differential analysis. The minimum number of cells to create the communication network for a cell population was set to 10. Heatmaps showing the differential number of interactions or interaction strength among different cell populations across the two datasets were generated using the CellChat netVisual_heatmap function. We performed systems signaling pathway differential analyses using the CellChat function rankSimilarity, which identifies the signaling networks with larger difference based on their Euclidean distance in the shared two-dimensions space. Larger distance implies larger differences of the communication networks between two datasets in terms of either functional or structure similarity. The CellChat function netVisual_bubble was used to identify and visualize up-regulated signaling ligand-receptor pairs between cells and pathways of interest. Cell-cell communication pathways of interest were visualized with chord diagrams.

#### scBCR and scTCR immune repertoire analyses

FASTQ files containing VDJ sequences were processed using the BD Rhapsody Targeted Analysis Pipeline with V(D)J processing in the Seven Bridges Platform. This pipeline generated dominant contigs for each CellID–chain combination in the Adaptive Immune Receptor Repertoire (AIRR) rearrangement schema including cell identifiers, read and molecule counts, full trimmed contig nucleotide and amino acid sequence, framework and CDR region nucleotide and amino acid sequence, V, D, J, and C gene segments, full length and productive status. scBCR and scTCR datasets in AIRR format were analyzed using the R package *Immunarch*^76^ to obtain clonotype metrics including clonality, diversity (measured by Chao1), top 200 public (shared) BCR and TCR clonotypes, isotype frequencies, and CDR lengths from each sample in clinical trial cohort. Somatic hypermutation levels(including silent and non-silent mutations) per unique IGHV-D-J region per isotype calculated over the CDR1/2 and FWR regions for each individual sample using the observedMutation function within the SHhazaM package.^77^ Multiple Wilcoxon tests with false discovery rate (FDR) Benjamini-Hochberg corrections were performed to determine BCR and TCR clonotypes altered by vitamin D. EnhancedVolcano^78^ was used to generate volcano plots depicting differential expressed BCR and TCR clonotypes with vitamin D. The R package Qgraph^74^ was used to generate correlation network plots depicting association of BCR and TCR clonotypes with Ig-bound gut bacteria.

### Quantification and statistical analysis

Bioinformatics analyses were executed in R (v4.4.0); data are expressed as mean ± standard error of the mean. Statistical analyses and visualizations were performed with GraphPad Prism (v10) and R (v4.40). Between-group differences were evaluated by paired two-tailed t-tests; multiple comparisons were assessed by one-way analysis of variance with Tukey’s post-hoc test or multiple Wilcoxon tests with false discovery rate (FDR) Benjamini-Hochberg where appropriate. Correlational analyses were performed using Spearman’s rank correlation analysis. Significance levels were denoted as ∗*p* < 0.05, ∗∗*p* < 0.01, ∗∗∗*p* < 0.001, and ∗∗∗∗*p* < 0.0001. Results with *p* < 0.05 were considered statistically significant.

### Additional resources

The trial was registered at clinicaltrials.gov (Unique identifier: NCT04828031).

## Supplemental information

Document S1. Figures S1–S12 and Tables S1, S4, S6, and S8

Table S2. Top 200 BCR clonotypes after vitamin D in IBD (Excel sheet), related to Figure 4

Table S3. Top 200 TCR clonotypes after vitamin D in IBD (Excel sheet), related to Figure 4

Table S5. TCR clonotype and Ig-seq correlations (Excel sheet), related to Figure 4

Table S7. Association of α4β7+ T cells and Ig-bound gut bacteria (Excel sheet), related to Figure 6

Document S2. Article plus supplemental information
