# Supplementary material for: Multi-omics reveal vitamin D regulation of immune-gut microbiome interactions and tolerogenic pathways in inflammatory bowel disease
Source: Cell Rep Med. 2026 Mar 26;7(4):102703. doi: 10.1016/j.xcrm.2026.102703 (PMC13130636; doi:10.1016/j.xcrm.2026.102703)
Supplement: Document S1. Figures S1–S12 and Tables S1, S4, S6, and S8 [file mmc1.pdf]

Cell Reports Medicine, Volume 7

Supplemental information

Multi-omics reveal vitamin D regulation of immune-gut microbiome interactions and tolerogenic pathways in inflammatory bowel disease

John Gubatan, Raoul S. Sojwal, Jiayu Ye, Theresa L. Boye, Jacqueline N. Hoang, Touran Fardeen, Michelle Temby, Samuel J.S. Rubin, Sean P. Spencer, Prasanti Kotagiri, Stephan Rogalla, Michael J. Rosen, Ole Haagen Nielsen, Scott Boyd, Justin Sonnenburg, and Sidhartha R. Sinha

Supplemental Figure and Figure legend

Figure S1

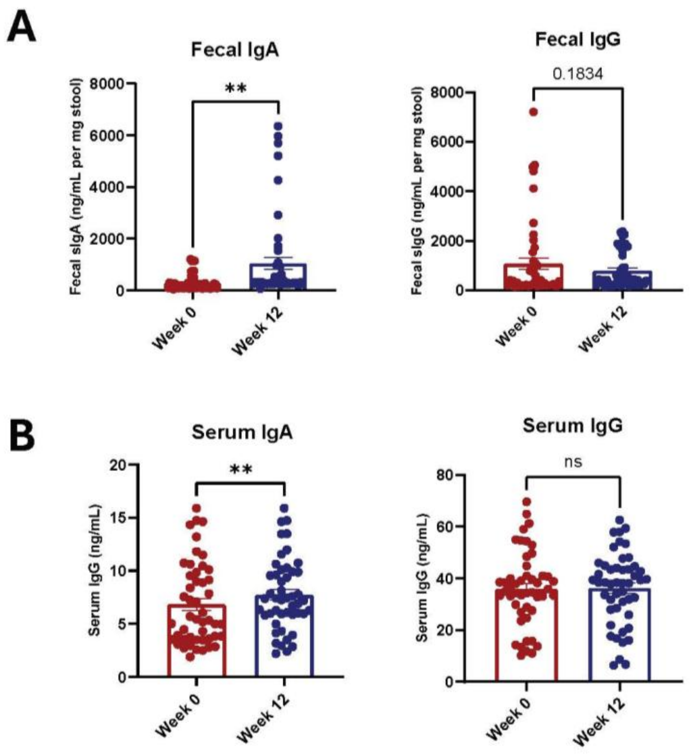

Figure S1. **Effects of vitamin D on secreted and circulating IgA and IgG. Related to Figure 1.** A) Secretory IgA and IgG measured by enzyme-linked immunosorbent essay (ELISA) from fecal supernatants. B) Serum IgA and IgG measured by ELISA (n=48 patients, two time points). Stars indicate paired T-tests p-values: non-significant (ns): $p > 0.05$; *: $p < 0.05$; ** $p < 0.01$; *** $p < 0.01$.

Figure S2

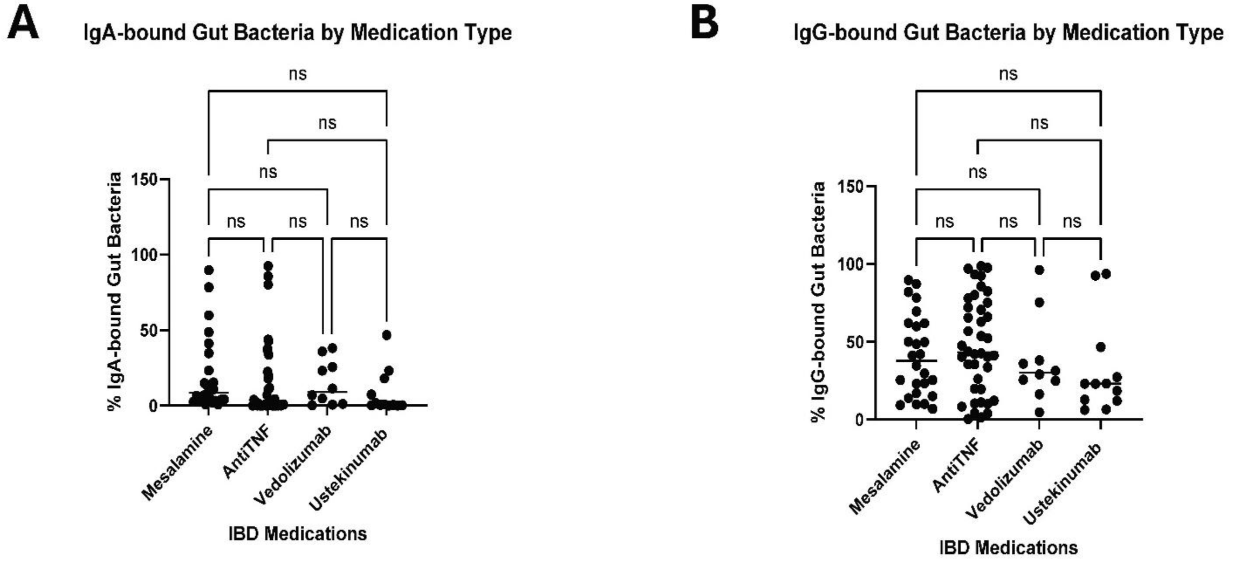

Figure S2. Sensitivity analyses on effects of IBD medication therapy class on immunoglobulin binding to gut bacteria in patients with IBD. **Related to Figure 1.** A) Multiple comparisons of IBD medication type and percentage of IgA-bound gut bacteria B) Multiple comparisons of IBD medication type and percentage of IgG-bound gut bacteria. Stars indicate paired T-tests p-values: non-significant (ns): $p > 0.05$; *: $p < 0.05$; ** $p < 0.01$; *** $p < 0.01$.

Figure S3

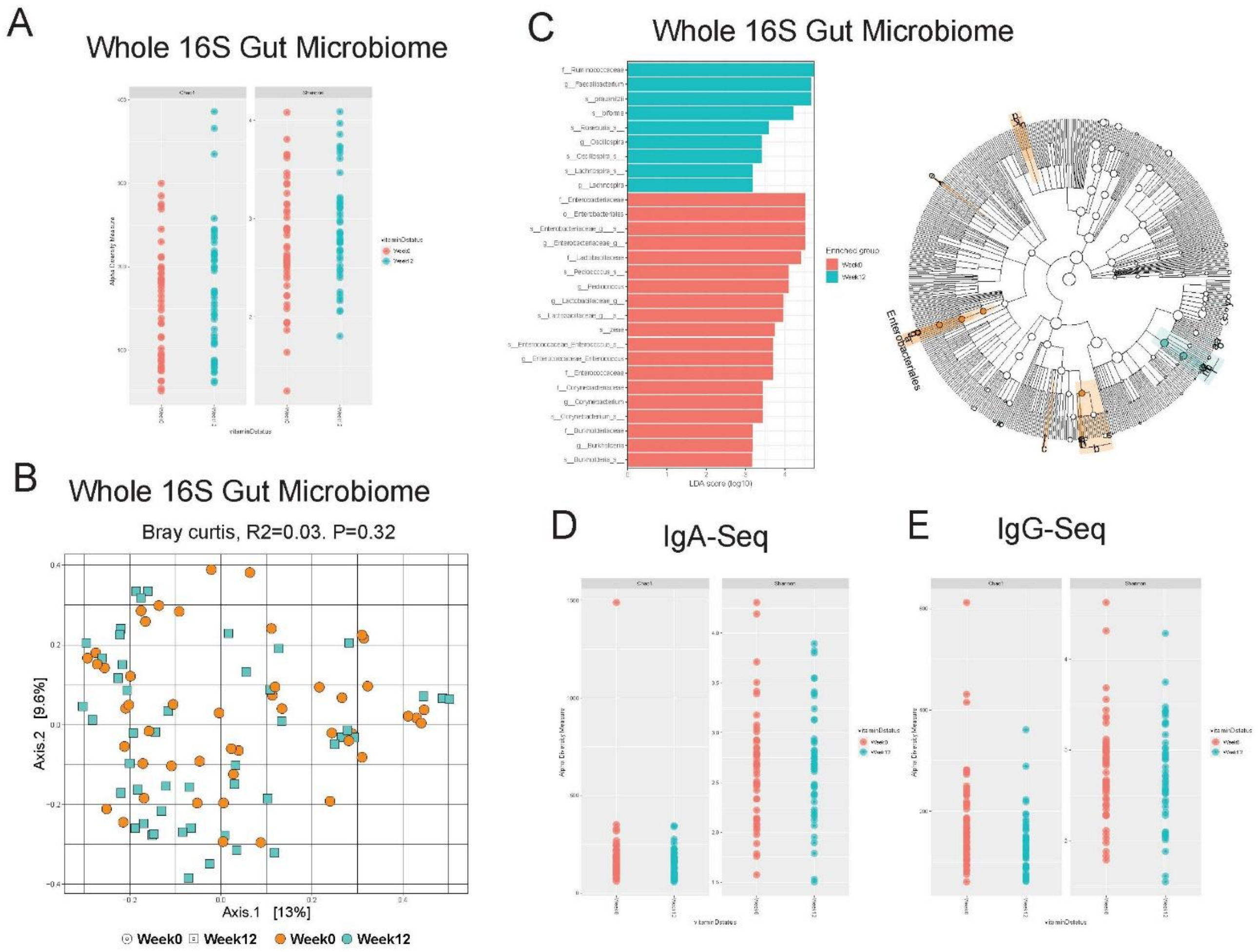

Figure S3. Effects of vitamin D on whole gut microbiome composition in patients with inflammatory bowel disease cells (n=48 patients, two time points). Related to Figure 2. A) Bar plots demonstrating no significant changes in alpha diversity measured by Chao1 and Shannon diversity index after vitamin D B)16S sequencing of whole gut microbiome reveals gut microbiota taxa significantly altered after 12 weeks of vitamin D by LEfSe linear discriminant analysis (LDA) effect size bar plot (left), LEfSe cladogram (right) C) Beta diversity by Bray curtis ordination plot of whole gut microbiome according to vitamin D status D) IgA-Seq alpha diversity bar plots E) IgG-Seq alpha diversity bar plots.

Figure S4

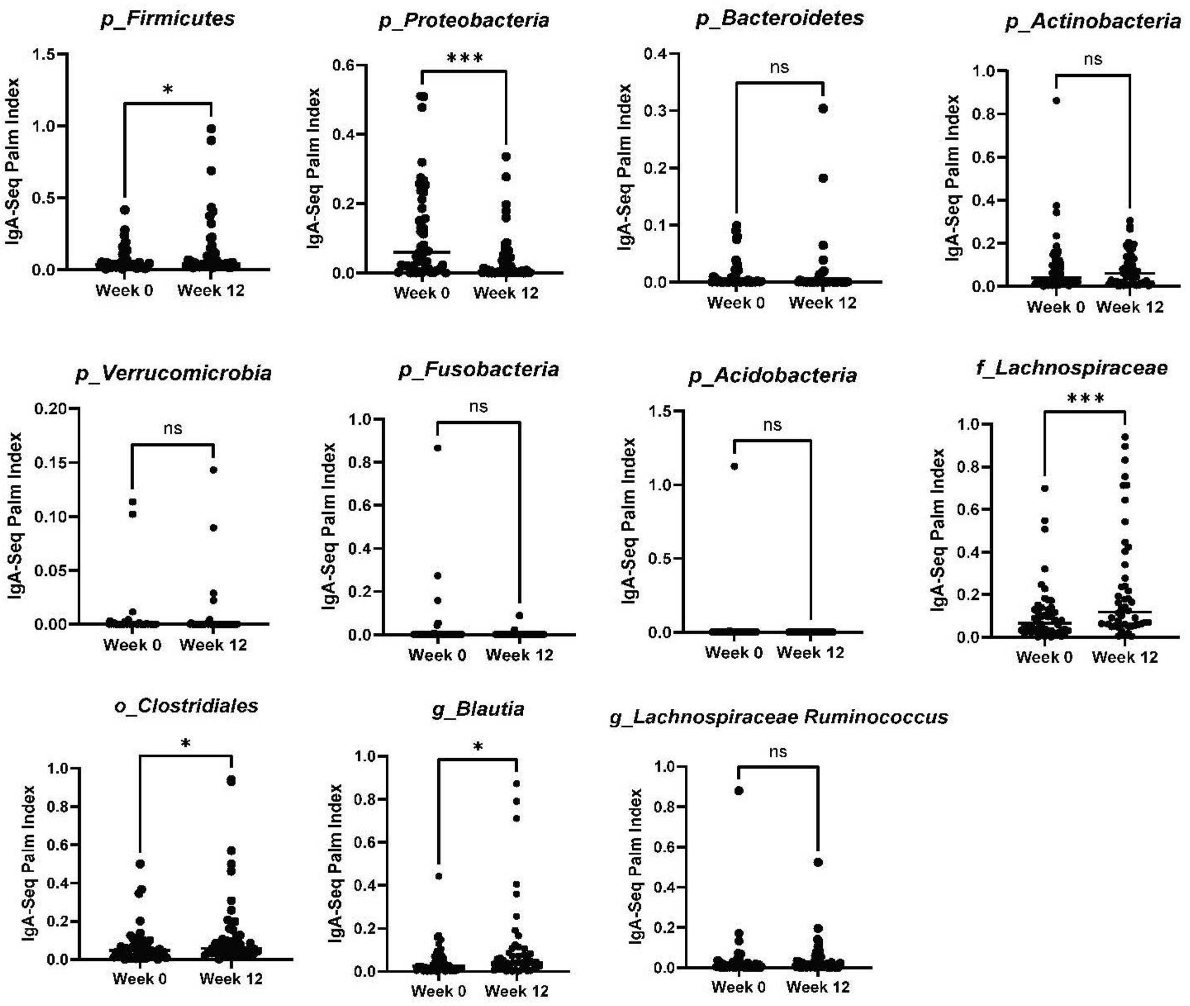

Figure S4. Effects of 12 weeks of vitamin D cells (n=48 patients, two time points) on IgA-binding to gut bacterial taxa (major phyla, select family, order, and genus) in patients with inflammatory bowel disease as measured by Palm Index. Related to Figure 2. Stars indicate paired T-tests p-values: ns: p > 0.05; *: p < 0.05; ** p < 0.01; *** p < 0.01.

Figure S5

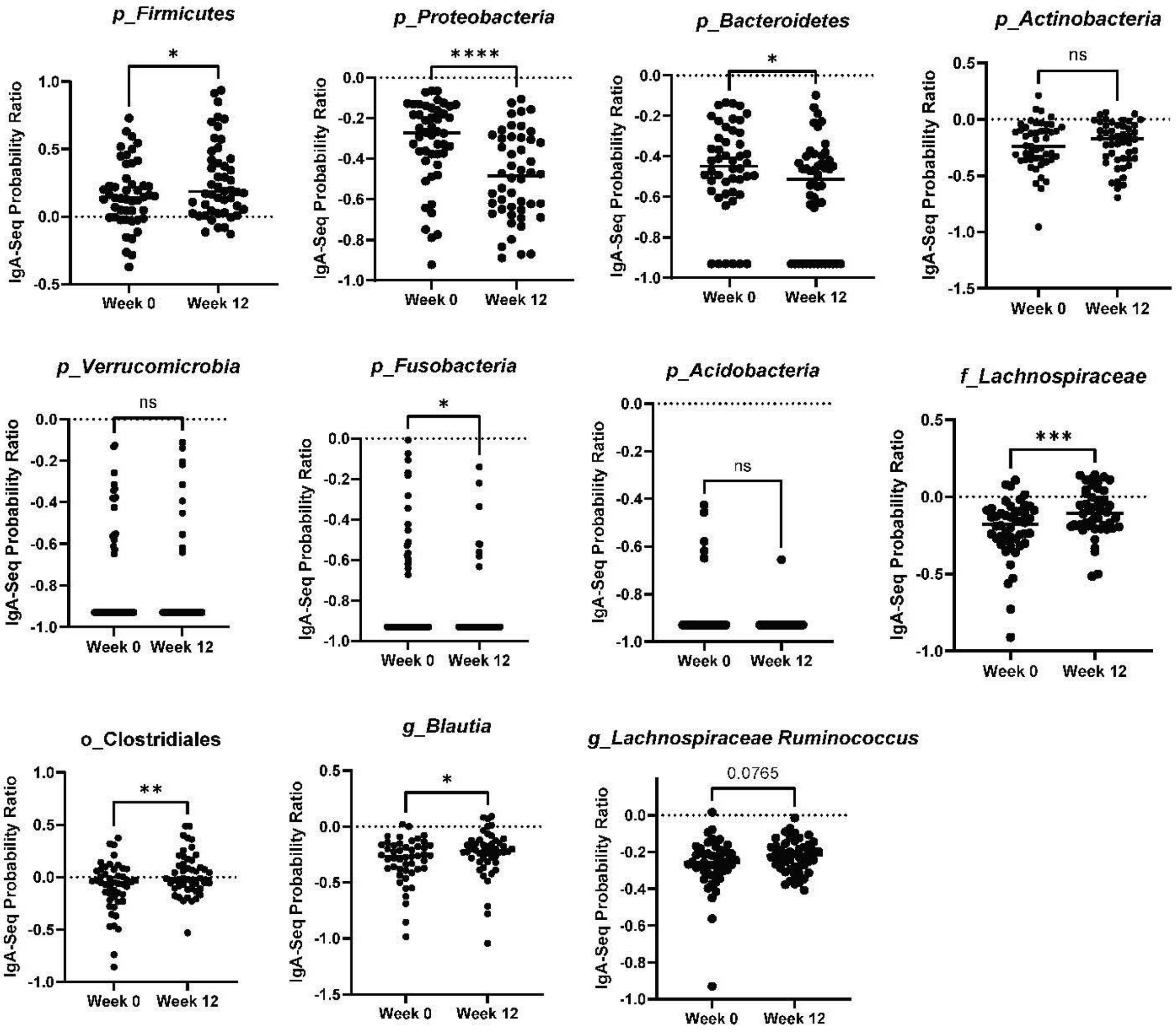

Figure S5. Effects of 12 weeks of vitamin D cells (n=48 patients, two time points) on IgA-binding to gut bacterial taxa (major phyla, select family, order, and genus) in patients with inflammatory bowel disease as measured by IgA-Seq probability ratio. **Related to Figure 2.** Stars indicate paired T-tests p-values: ns: $p > 0.05$; *: $p < 0.05$; ** $p < 0.01$; *** $p < 0.01$.

Figure S6

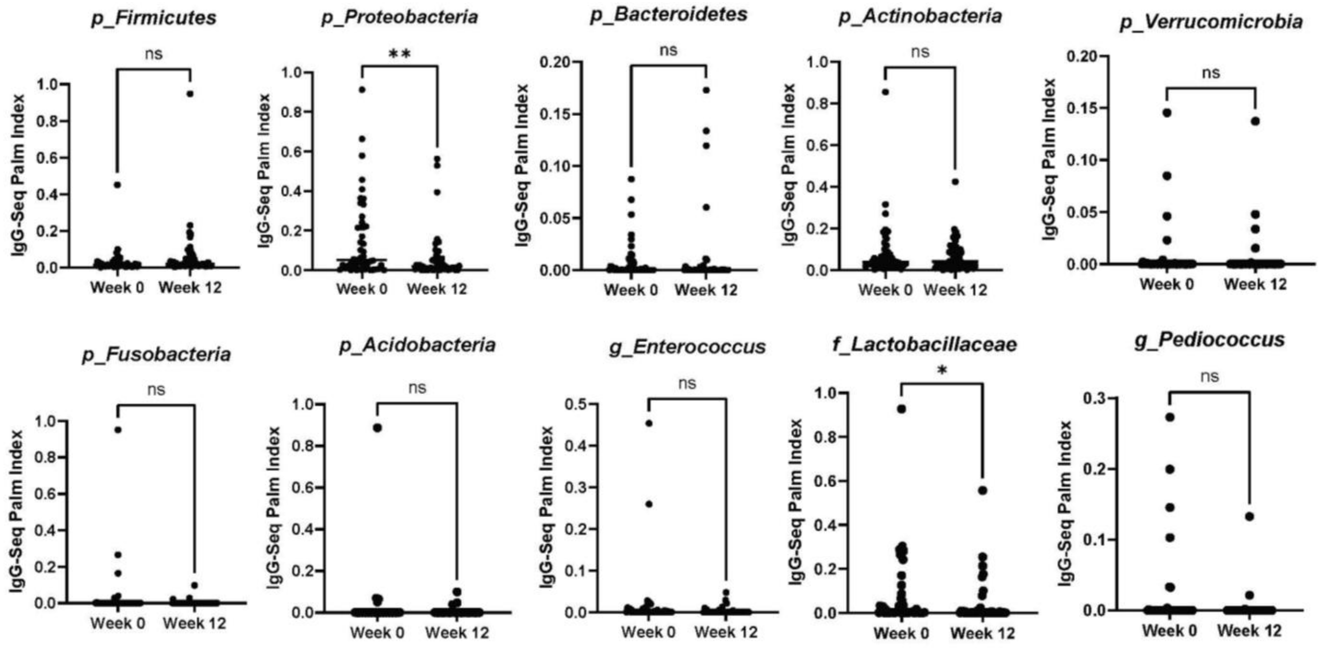

Figure S6. Effects of 12 weeks of vitamin D cells (n=48 patients, two time points) on IgG-binding to gut bacterial taxa (major phyla, select family and genus) in patients with inflammatory bowel disease as measured by Palm Index. Related to Figure 2. Stars indicate paired T-tests p-values: ns: $p > 0.05$; *: $p < 0.05$; ** $p < 0.01$; *** $p < 0.01$.

Figure S7

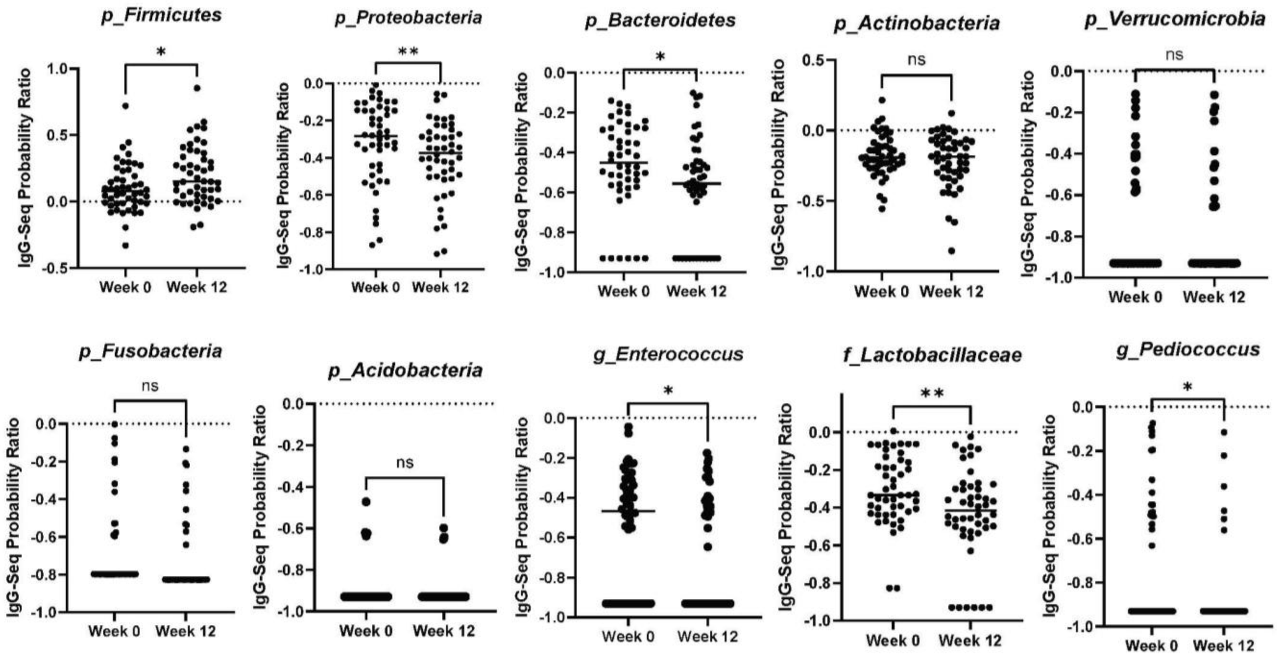

Figure S7. Effects of 12 weeks of vitamin D cells (n=48 patients, two time points) on IgG-binding to gut bacterial taxa (major phyla, select family and genus) in patients with inflammatory bowel disease as measured by IgG-Seq probability ratio. Related to Figure 2. Stars indicate paired T-tests p-values: ns: p > 0.05; *: p < 0.05; ** p < 0.01; *** p < 0.01.

Figure S8

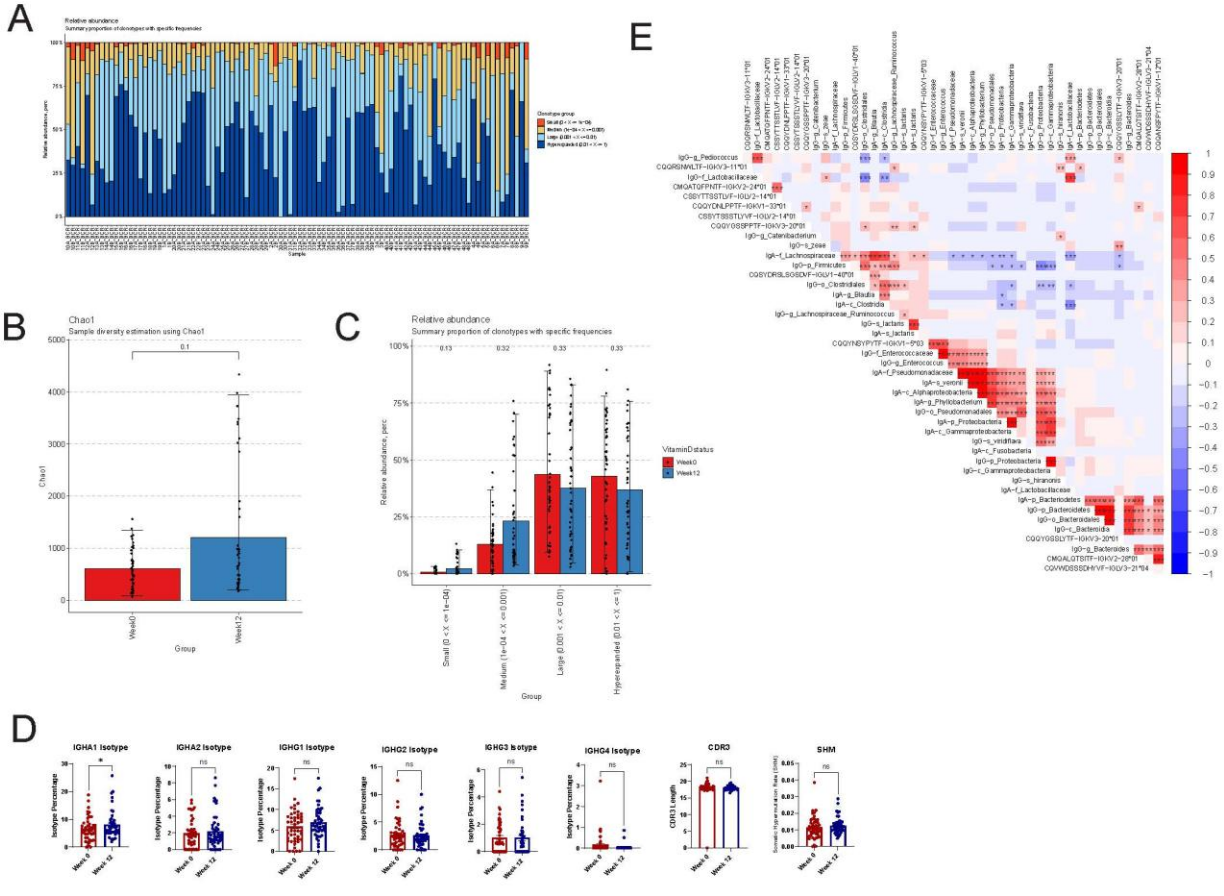

Figure S8. Effects of vitamin D cells (n=48 patients, two time points) on BCR repertoire. Related to Figure 4. A) Bar plot summary of BCR clonotype relative abundance by patient sample and clonotype group. B) BCR clonal diversity estimated by Chao1 before and after 12 weeks of vitamin D C) Relative abundance of BCR clonotypes by vitamin D status and clonotype group. D) Effects of vitamin D on BCR metrics (isotype frequency, complementarity-determining region (CDR3) length, somatic hypermutation rates). E) Correlation matrix demonstrating association of BCR clonotypes altered by vitamin D and IgA- and IgG-bound gut microbiota taxa. Stars indicate nominal Wilcoxon signed-rank test p-values: ns: p > 0.05; *: p < 0.05; ** p < 0.01; *** p < 0.01.

Figure S9

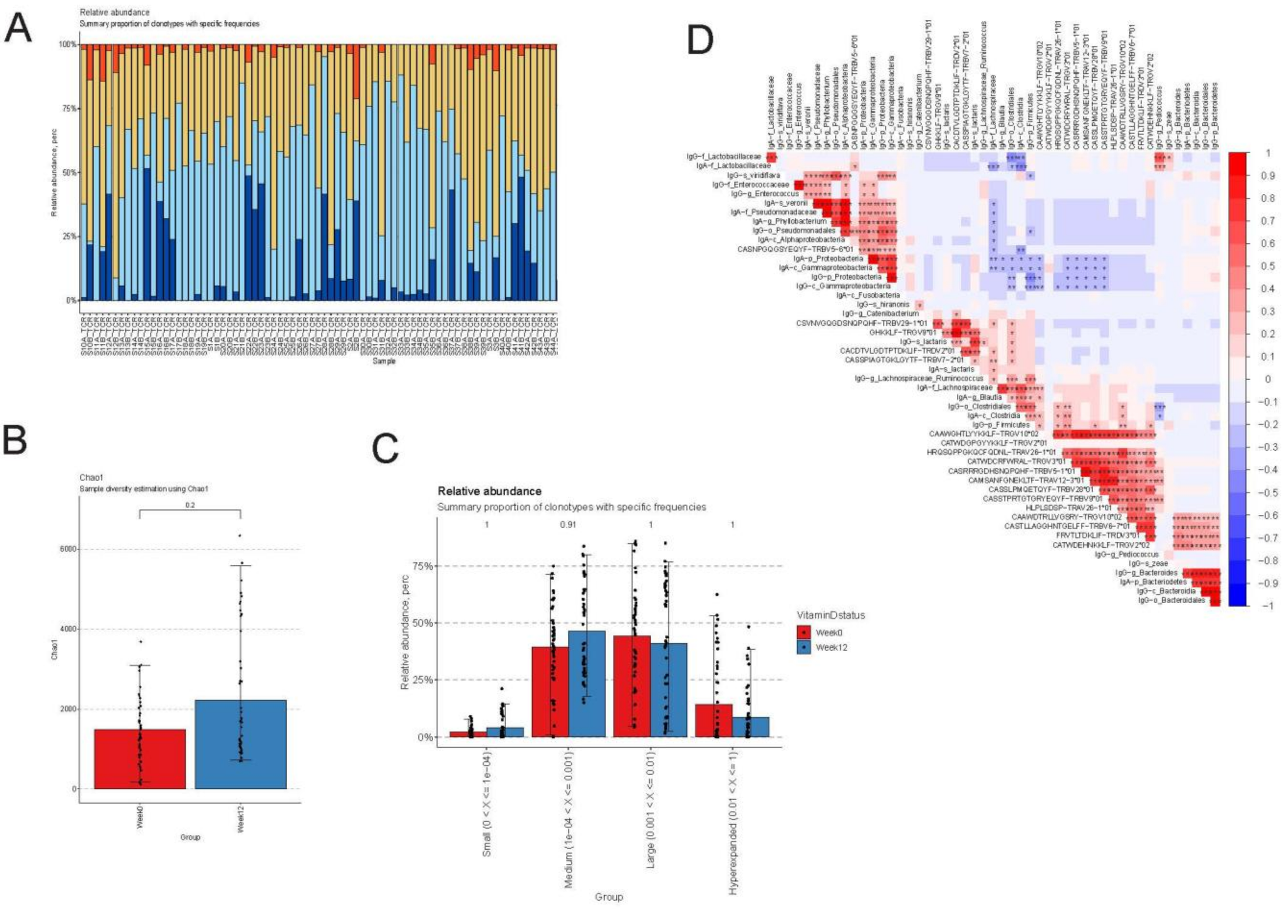

Figure S9. Effects of vitamin D cells (n=48 patients, two time points) on TCR repertoire. Related to Figure 4. A) Bar plot summary of TCR clonotype relative abundance by patient sample and clonotype group. B) TCR clonal diversity estimated by Chao1 before and after 12 weeks of vitamin D C) Relative abundance of TCR clonotypes by vitamin D status and clonotype group. taxa D) Correlation matrix demonstrating association of TCR clonotypes altered by vitamin D and IgA- and IgG-bound gut microbiota taxa. Stars indicate nominal Wilcoxon signed-rank test p-values: ns: $p > 0.05$; *: $p < 0.05$; ** $p < 0.01$; *** $p < 0.01$.

Figure S10

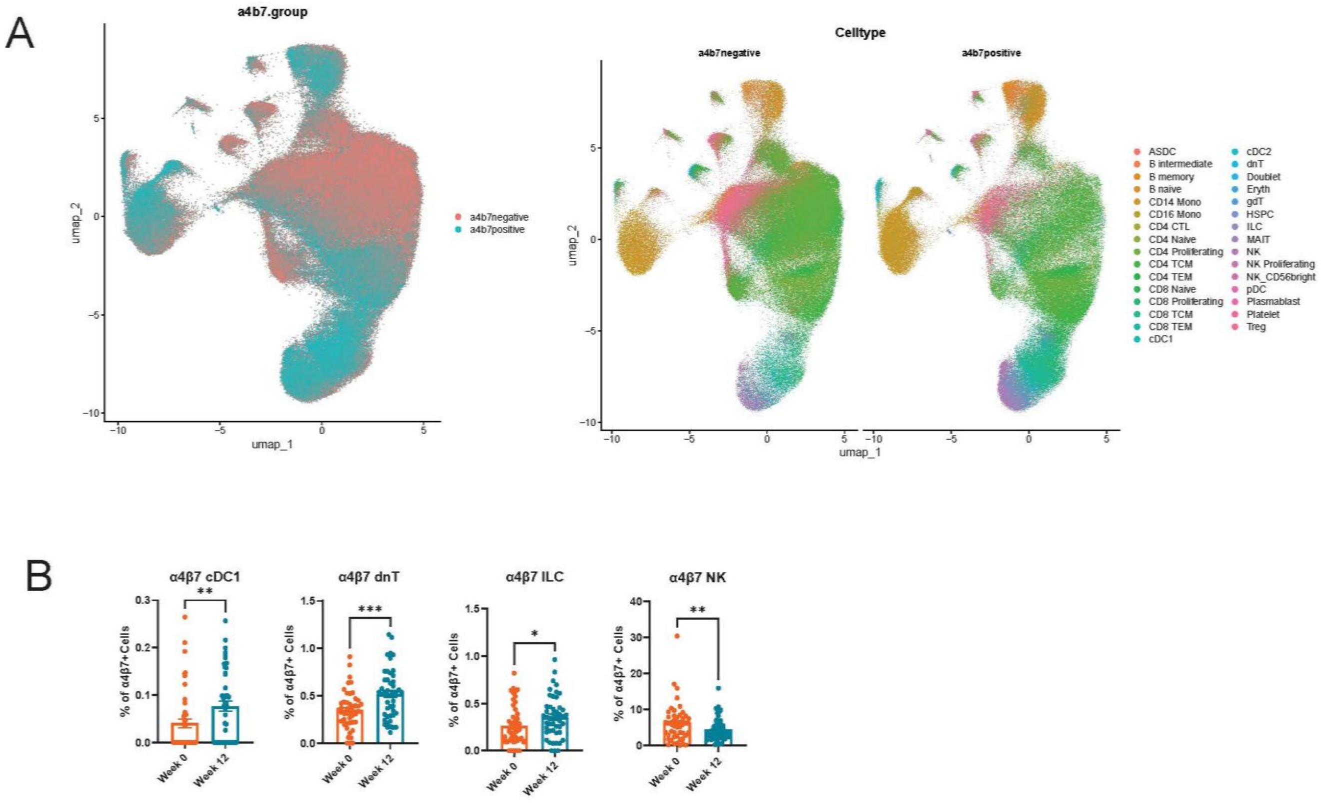

Figure S10. a4β7+ PBMC scRNA-seq. Related to Figure 5 and 6. A) UMAP of PBMC scRNA-seq atlas cells (n=48 patients, two time points) according to a4β7 expression status (left) and cell type split by a4β7 expression status (right). B) Differential abundance analyses reveal a4β7+ immune cell types regulated by vitamin D.

Figure S11

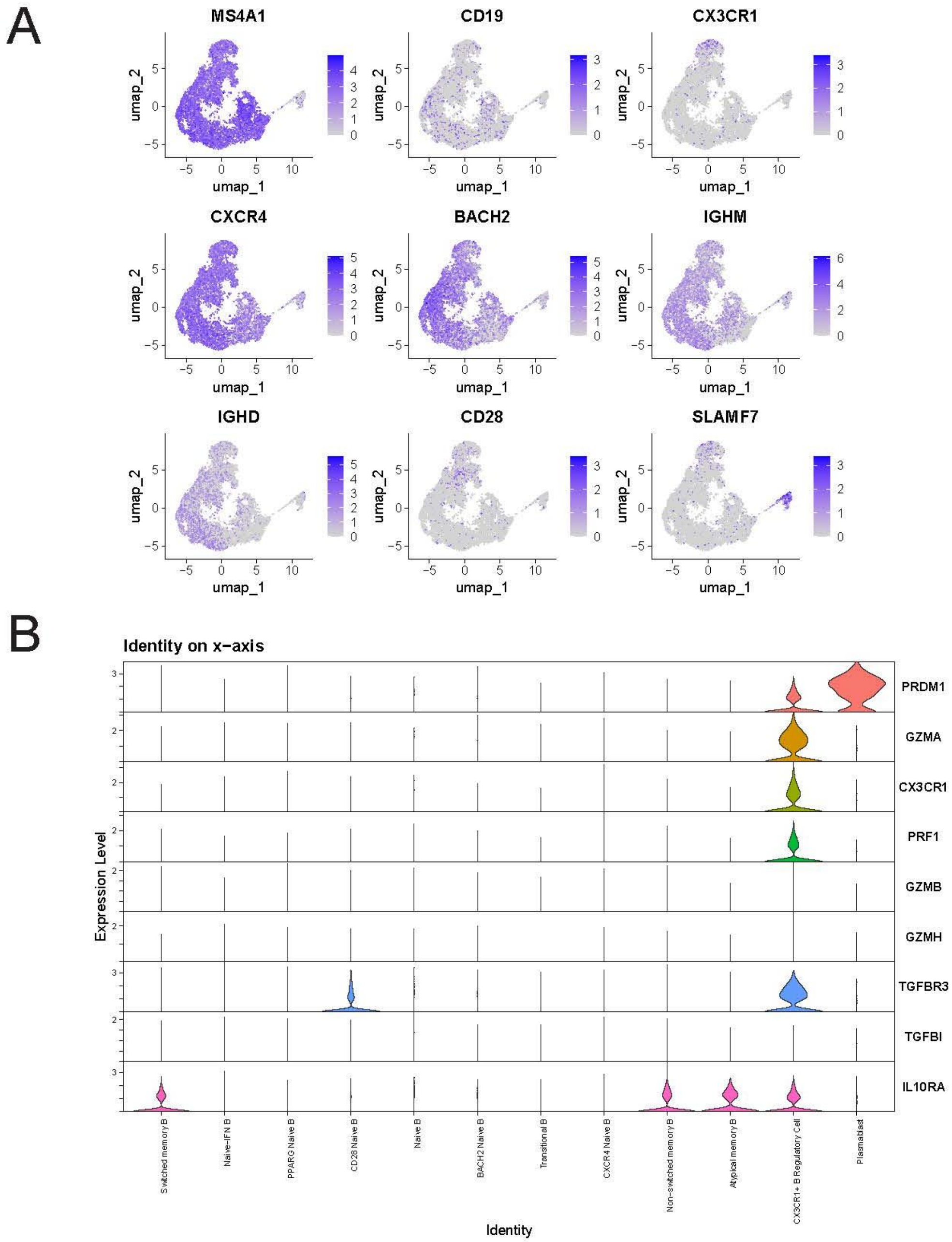

Figure S11. a4β7+ B Cells. Related to Figure 5. A) Feature Plot demonstrating representative B cell markers by cluster B) Violin Plot demonstrating B regulatory cell markers (*CX3CR1, PRF1, GZMA, GZMB, GZMH, TGFBR3, PRDM1, IL10RA, TGFBI)* according to B cell subset.

Figure S12

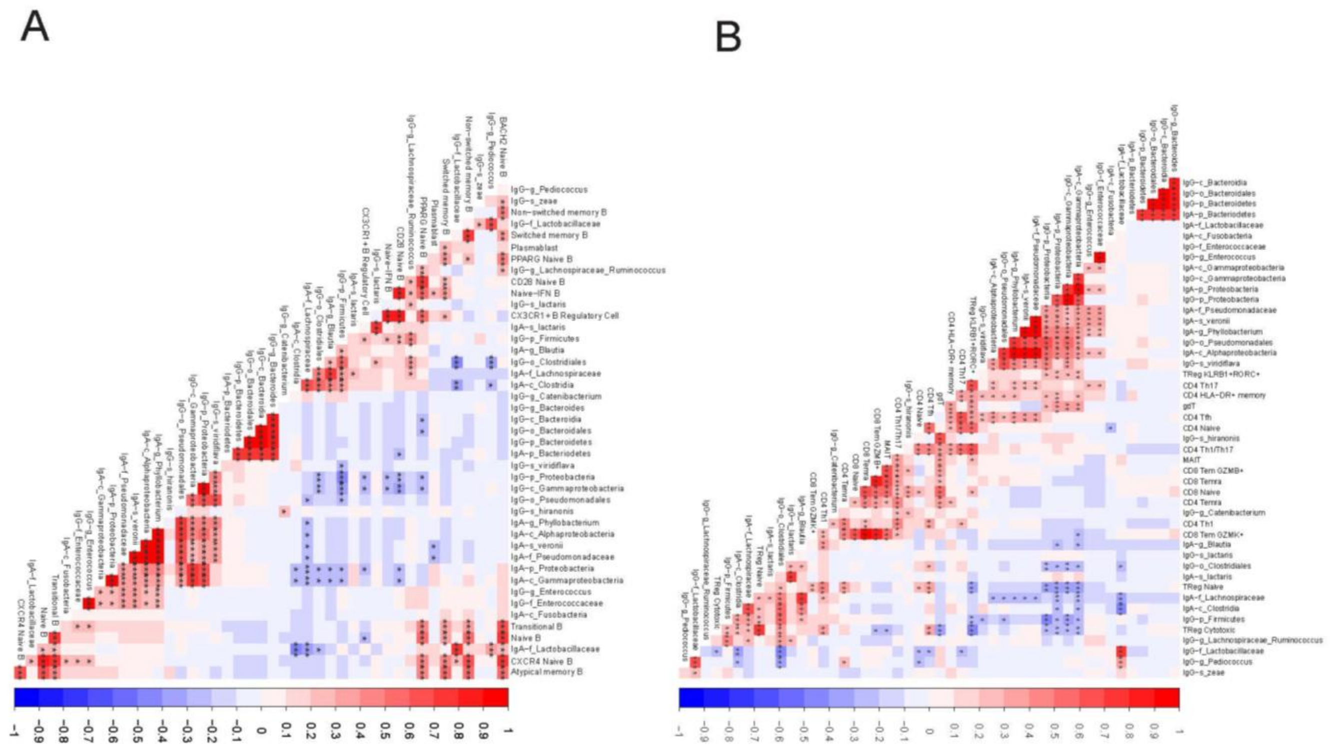

Figure S12. a4β7+ B and T Cell Ig-Seq Correlation Matrices. Related to Figure 5 and 6. A) Correlation matrix demonstrating association of a4β7+ B Cells altered by vitamin D and IgA- and IgG-bound gut microbiota taxa. B) Correlation matrix demonstrating association of a4β7+ T Cells altered by vitamin D and IgA- and IgG-bound gut microbiota taxa. Stars indicate nominal Wilcoxon signed-rank test p-values: ns: $p > 0.05$; *: $p < 0.05$; ** $p < 0.01$; *** $p < 0.01$.

Table S1. IgA- and IgG-bound Gut Bacteria and IBD Medication Class. Related to Figure 1.

Table S1. IgA- and IgG-bound Gut Bacteria and IBD Medication Class

IGA-SEQ

Tukey's multiple comparisons test	Mean 1	Mean 2	Mean diff.	95.00% CI of diff.	q	Adjusted P Value
Mesalamine vs. AntiTNF	19.54	14.03	5.511	-9.388 to 20.41	1.371	0.767
Mesalamine vs. Vedolizumab	19.54	14.75	4.791	-17.22 to 26.80	0.807	0.9406
Mesalamine vs. Ustekinumab	19.54	8.259	11.28	-9.362 to 31.92	2.026	0.483
AntiTNF vs. Vedolizumab	14.03	14.75	-0.7204	-21.63 to 20.19	0.1277	0.9997
AntiTNF vs. Ustekinumab	14.03	8.259	5.767	-13.70 to 25.23	1.098	0.8649
Vedolizumab vs. Ustekinumab	14.75	8.259	6.487	-18.84 to 31.81	0.9496	0.9076

IGG-SEQ

Tukey's multiple comparisons test	Mean 1	Mean 2	Mean diff.	95.00% CI of diff.	q	Adjusted P Value
Mesalamine vs. AntiTNF	40.97	47.73	-6.763	-25.98 to 12.45	1.305	0.7928
Mesalamine vs. Vedolizumab	40.97	37.72	3.253	-25.13 to 31.63	0.425	0.9905
Mesalamine vs. Ustekinumab	40.97	32.12	8.849	-17.77 to 35.46	1.232	0.8195
AntiTNF vs. Vedolizumab	47.73	37.72	10.02	-16.95 to 36.98	1.377	0.7647
AntiTNF vs. Ustekinumab	47.73	32.12	15.61	-9.489 to 40.71	2.306	0.3674
Vedolizumab vs. Ustekinumab	37.72	32.12	5.596	-27.06 to 38.25	0.6353	0.9696

Table S4. BCR Clonotype and Ig-Seq Correlations. Related to Figure 4.

Table S4. BCR Clonotype Ig-Seq Correlations

BCR Clonotype	Ig-Seq	Pearson R	P Value
CQQYNSYPYTF-IGKV1-5*03	IgG-g_Enterococcus	0.618	3.275E-11
CQQYNSYPYTF-IGKV1-5*03	IgG-f_Enterococcaceae	0.618	3.280E-11
CMQALQTSITF-IGKV2-28*01	IgA-p_Bacteriodetes	0.607	9.081E-11
CMQALQTSITF-IGKV2-28*01	IgG-g_Bacteroides	0.559	4.838E-09
CQQANSFPYTF-IGKV1-12*01	IgA-p_Bacteriodetes	0.551	8.454E-09
CQQANSFPYTF-IGKV1-12*01	IgG-g_Bacteroides	0.550	9.044E-09
CMQALQTSITF-IGKV2-28*01	IgG-c_Bacteroidia	0.473	1.498E-06
CMQALQTSITF-IGKV2-28*01	IgG-o_Bacteroidales	0.473	1.499E-06
CMQALQTSITF-IGKV2-28*01	IgG-p_Bacteroidetes	0.439	9.659E-06
CQQANSFPYTF-IGKV1-12*01	IgG-c_Bacteroidia	0.415	3.220E-05
CQQANSFPYTF-IGKV1-12*01	IgG-o_Bacteroidales	0.415	3.220E-05
CQQANSFPYTF-IGKV1-12*01	IgG-p_Bacteroidetes	0.382	1.460E-04
CQSYDRSLSGSDVF-IGLV1-40*01	IgA-g_Blautia	0.349	5.619E-04
CQQYGSSLYTF-IGKV3-20*01	IgG-s_zeae	0.327	1.317E-03
CQVWDSSSDHYVF-IGLV3-21*04	IgG-g_Bacteroides	0.304	2.884E-03
CQQYGSSPPTF-IGKV3-20*01	IgG-g_Lachnospiraceae_Ruminococcus	0.278	6.580E-03
CQQRSNWLTF-IGKV3-11*01	IgG-s_hiranonis	0.274	7.469E-03
CQQYGSSLYTF-IGKV3-20*01	IgG-g_Pediococcus	0.260	1.138E-02
CQVWDSSSDHYVF-IGLV3-21*04	IgG-p_Bacteroidetes	0.253	1.382E-02
CQQRSNWLTF-IGKV3-11*01	IgG-g_Lachnospiraceae_Ruminococcus	0.249	1.552E-02
CQQRSNWLTF-IGKV3-11*01	IgA-p_Bacteriodetes	0.242	1.883E-02
CQVWDSSSDHYVF-IGLV3-21*04	IgG-o_Bacteroidales	0.238	2.114E-02
CQVWDSSSDHYVF-IGLV3-21*04	IgG-c_Bacteroidia	0.238	2.114E-02
CQSYDRSLSGSDVF-IGLV1-40*01	IgA-f_Lachnospiraceae	0.229	2.653E-02
CQQYNSYPYTF-IGKV1-5*03	IgA-f_Lachnospiraceae	0.216	3.662E-02
CQQYGSSPPTF-IGKV3-20*01	IgA-s_lactaris	0.214	3.840E-02
CQQYGSSPPTF-IGKV3-20*01	IgG-o_Clostridiales	0.213	3.896E-02
CQQYGSSLYTF-IGKV3-20*01	IgA-f_Lachnospiraceae	-0.207	4.576E-02
CQQYGSSLYTF-IGKV3-20*01	IgG-p_Firmicutes	-0.238	2.084E-02

Table S6. Association of α4β7+ B Cells and Ig-bound Gut Bacteria. Related to Figure 5.

Table S6. Association of α4β7+ B Cells and Ig-bound Gut Bacteria			
**α4β7+ B Cell Subtype**	**Ig-Seq**	**Pearson R**	**P Value**
PPARG Naive B	IgG-g_Lachnospiraceae_Ruminococcus	0.286	4.738E-03
CD28 Naive B	IgG-p_Firmicutes	0.277	6.381E-03
CX3CR1+ B Regulatory Cell	IgG-p_Firmicutes	0.261	1.030E-02
Naive-IFN B	IgG-p_Firmicutes	0.253	1.274E-02
CXCR4 Naive B	IgA-f_Lactobacillaceae	0.253	1.287E-02
Transitional B	IgA-f_Lactobacillaceae	0.238	1.935E-02
CD28 Naive B	IgG-g_Lachnospiraceae_Ruminococcus	0.234	2.200E-02
Naive B	IgA-f_Lactobacillaceae	0.233	2.263E-02
BACH2 Naive B	IgG-s_zeae	0.232	2.311E-02
CXCR4 Naive B	IgA-c_Fusobacteria	0.229	2.492E-02
Transitional B	IgG-f_Enterococcaceae	0.224	2.789E-02
Transitional B	IgG-g_Enterococcus	0.224	2.791E-02
BACH2 Naive B	IgG-g_Lachnospiraceae_Ruminococcus	0.216	3.483E-02
BACH2 Naive B	IgA-f_Lactobacillaceae	0.215	3.503E-02
Naive-IFN B	IgG-g_Lachnospiraceae_Ruminococcus	0.206	4.436E-02
CXCR4 Naive B	IgG-f_Lactobacillaceae	0.205	4.484E-02
CXCR4 Naive B	IgG-f_Enterococcaceae	0.202	4.795E-02
CXCR4 Naive B	IgG-g_Enterococcus	0.202	4.806E-02
CD28 Naive B	IgA-p_Bacteriodetes	-0.207	4.342E-02
CX3CR1+ B Regulatory Cell	IgA-p_Proteobacteria	-0.208	4.244E-02
PPARG Naive B	IgG-c_Bacteroidia	-0.208	4.164E-02
PPARG Naive B	IgG-o_Bacteroidales	-0.208	4.164E-02
Naive-IFN B	IgG-c_Gammaproteobacteria	-0.216	3.459E-02
Plasmablast	IgA-s_veronii	-0.219	3.167E-02
Plasmablast	IgA-f_Pseudomonadaceae	-0.220	3.136E-02
Naive-IFN B	IgG-p_Proteobacteria	-0.222	2.937E-02
PPARG Naive B	IgG-c_Gammaproteobacteria	-0.239	1.927E-02
CD28 Naive B	IgA-c_Gammaproteobacteria	-0.241	1.806E-02
PPARG Naive B	IgG-p_Proteobacteria	-0.242	1.775E-02
CD28 Naive B	IgA-p_Proteobacteria	-0.250	1.385E-02
CX3CR1+ B Regulatory Cell	IgG-c_Gammaproteobacteria	-0.256	1.195E-02
CX3CR1+ B Regulatory Cell	IgG-p_Proteobacteria	-0.257	1.155E-02
CD28 Naive B	IgG-p_Proteobacteria	-0.293	3.804E-03
CD28 Naive B	IgG-c_Gammaproteobacteria	-0.308	2.290E-03

Table S8. IBD Patient Cohort Metadata. Related to Table 1.

Table SS8. IBD Patient Cohort Metadata			
**Patient ID**	**Age**	**Sex**	**IBD Subtype**
S1	36	M	Ulcerative Colitis
S2	27	F	Ulcerative Colitis
S3	24	M	Ulcerative Colitis
S4	25	M	Ulcerative Colitis
S5	42	F	Ulcerative Colitis
S6	41	M	Ulcerative Colitis
S7	37	M	Ulcerative Colitis
S8	51	F	Ulcerative Colitis
S9	75	M	Crohn's Disease
S10	53	M	Ulcerative Colitis
S11	26	M	Crohn's Disease
S12	34	F	Ulcerative Colitis
S13	58	F	Ulcerative Colitis
S14	37	F	Crohn's Disease
S15	38	F	Ulcerative Colitis
S16	46	M	Ulcerative Colitis
S17	37	M	Ulcerative Colitis
S18	24	M	Crohn's Disease
S19	47	F	Crohn's Disease
S20	33	F	Crohn's Disease
S21	22	F	Crohn's Disease
S22	21	F	Ulcerative Colitis
S23	41	F	Ulcerative Colitis
S24	32	M	Ulcerative Colitis
S25	32	M	Ulcerative Colitis
S26	39	F	Ulcerative Colitis
S27	67	F	Crohn's Disease
S28	61	F	Ulcerative Colitis
S29	36	M	Ulcerative Colitis
S30	46	M	Ulcerative Colitis
S31	34	M	Ulcerative Colitis
S32	30	M	Ulcerative Colitis
S33	23	F	Crohn's Disease
S34	33	M	Crohn's Disease
S35	29	F	Crohn's Disease
S36	48	F	Crohn's Disease
S37	39	F	Crohn's Disease

S38	30	F	Crohn's Disease
S39	47	M	Ulcerative Colitis
S40	38	M	Crohn's Disease
S41	76	F	Crohn's Disease
S42	31	M	Crohn's Disease
S43	42	F	Crohn's Disease
S44	55	F	Ulcerative Colitis
S45	22	F	Crohn's Disease
S46	36	M	Crohn's Disease
S47	43	F	Ulcerative Colitis
S48	26	F	Crohn's Disease
